# Supplementary material for: Paralogs of Slitrk cell adhesion molecules configure excitatory synapse specificity via distinct cellular mechanisms
Source: PLoS Biol. 2025 Dec 18;23(12):e3003576. doi: 10.1371/journal.pbio.3003576 (PMC12742733; doi:10.1371/journal.pbio.3003576)
Supplement: S1 Fig — (A) Strategy used to generate Slitrk1-cKO mice. LoxP sites were introduced at positions flanking the neomycin gene, FLP recombinant target (FRT), and exon 1 (E1) of the murine Slitrk1 gene. Black arrows indicate forward and reverse primers used for genotyping. Note that lacZ and neomycin cassettes are two separate markers. (B) PCR genotyping of Slitrk1-floxed mice. The band size of the Slitrk1-floxed allele was 368 bp. (C) Quantitative RT-PCR analyses of Slitrk mRNA levels in the hippocampal lysates of Slitrk1-cKO mice. Data are presented as means ± SEMs (n = 3 mice/group). (D) Immunohistochemical validation of commercially available anti-Slitrk1 and anti-Slitrk2 antibodies using brain slices from Slitrk1-cKO (left) and Slitrk2-cKO (right) mice, respectively. The indicated Slitrk floxed mice were stereotactically injected with AAVs expressing Cre recombinase or inactive Cre (ΔCre; control), with immunohistochemical analyses performed 2 weeks later. Scale bar, 50 μm (applies to all images). Abbreviations: SO, stratum oriens; SP, stratum pyramidale; SR, stratum radiatum. (E and F) Representative immunoblots (E) of hippocampal lysates from Slitrk1f/f, Slitrk2f/f, Slitrk2 V89Mf/f, and Slitrk1/2f/f mice expressing Cre or ΔCre (control). Summary graphs (F) showing levels of various synaptic proteins, analyzed by semi-quantitative immunoblotting. Data are presented as means ± SEMs (n = 4–8 mice/group; *p < 0.05, **p < 0.01, ***p < 0.001; two-tailed unpaired t test). A red asterisk indicates nonspecific bands. Numerical data can be found in S1 Data. S2 Fig. Validation of Slitrk1 and Slitrk2 antibodies used in eMAP tissues derived from Slitrk-cKO mice. (A and B) Immunohistochemical staining-based validation of the antibodies recognizing Slitrk1 (top) and Slitrk2 (bottom) with the postsynaptic markers, SHANK2 and PSD-95, in the SR (A) and SLM (B) regions of the CA1 hippocampus of ΔCre control and Cre cKO mice. Slitrk-floxed mice were stereotactically injected with AAVs expres [file pbio.3003576.s001.docx]

**Supplementary Figures and Tables for Kim, Kim and Kim et al., “Paralogs of Slitrk cell adhesion molecules configure excitatory synapse specificity via distinct cellular mechanisms”**

**SUPPLEMENTARY FIGURES AND FIGURE LEGENDS**

**
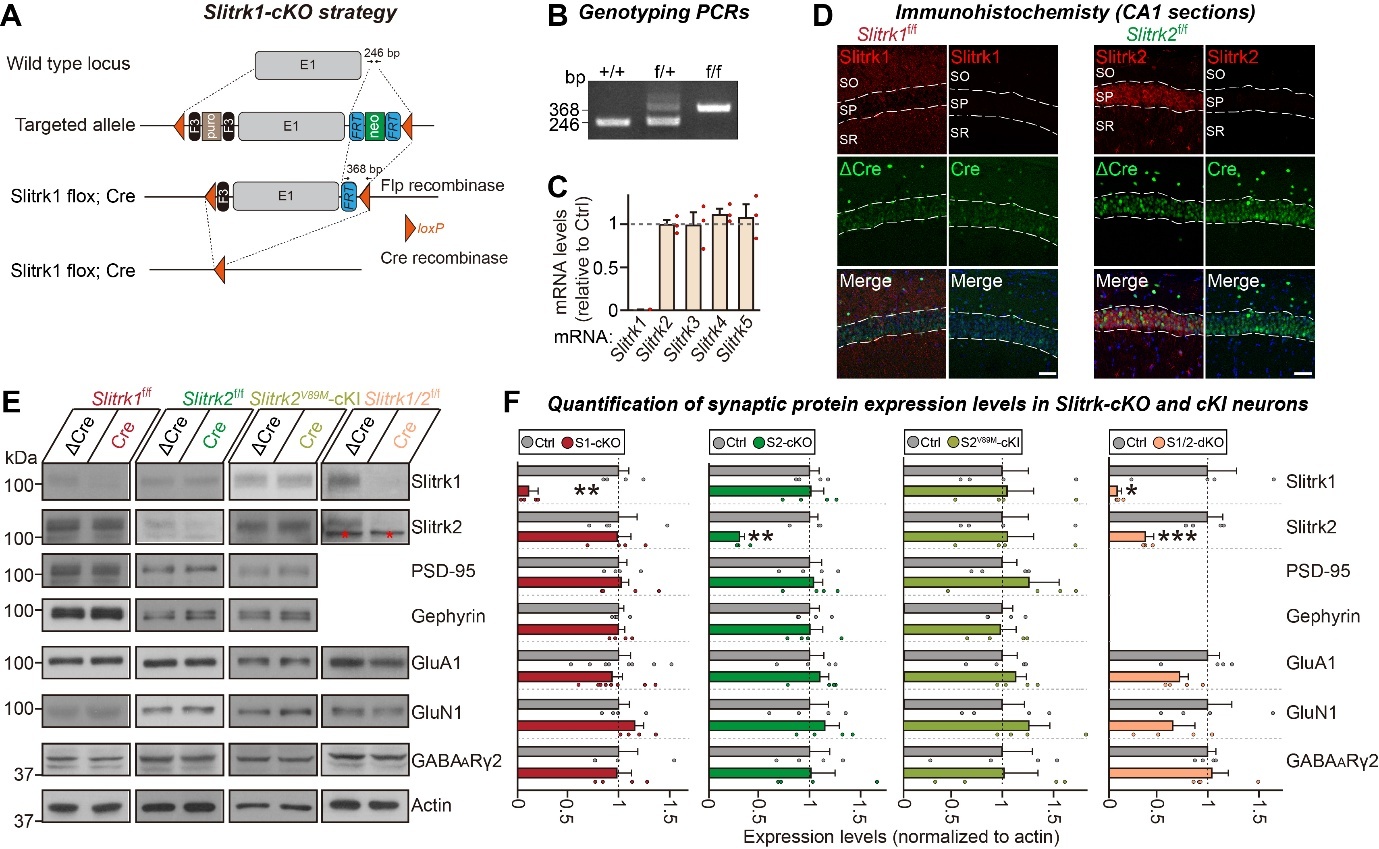
**

**S1 Fig. Validation of Slitrk1 and Slitrk2 antibodies used in the current study and analyses of the effects of conditional deletion of Slitrk proteins on synaptic protein expression.**

(**A**) Strategy used to generate *Slitrk1*-cKO mice. LoxP sites were introduced at positions flanking the neomycin gene, FLP recombinant target (FRT), and exon 1 (E1) of the murine *Slitrk1* gene. Black arrows indicate forward and reverse primers used for genotyping. Note that lacZ and neomycin cassettes are two separate markers.

(**B**) PCR genotyping of *Slitrk1*-floxed mice. The band size of the *Slitrk1*-floxed allele was 368 bp.

(**C**) Quantitative RT-PCR analyses of *Slitrk* mRNA levels in the hippocampal lysates of *Slitrk1*-cKO mice. Data are presented as means ± SEMs (n = 3 mice/group).

(**D**) Immunohistochemical validation of commercially available anti-Slitrk1 and anti-Slitrk2 antibodies using brain slices from *Slitrk1*-cKO (**left**) and *Slitrk2-*cKO (**right**) mice, respectively. The indicated *Slitrk* floxed mice were stereotactically injected with AAVs expressing Cre recombinase or inactive Cre (ΔCre; control), with immunohistochemical analyses performed 2 weeks later. Scale bar, 50 μm (applies to all images). Abbreviations: SO, *stratum oriens*; SP, *stratum pyramidale*; SR, *stratum radiatum*.

(**E** and **F**) Representative immunoblots (**E**) of hippocampal lysates from *Slitrk1*^f/f^, *Slitrk2*^f/f^, *Slitrk2* V89M^f/f^, and *Slitrk1/2*^f/f^ mice expressing Cre or ΔCre (control). Summary graphs (**F**) showing levels of various synaptic proteins, analyzed by semi-quantitative immunoblotting. Data are presented as means ± SEMs (n = 4–8 mice/group; **p* < 0.05, ***p* < 0.01, ****p* < 0.001; two-tailed unpaired t-test). A red asterisk indicates non-specific bands. Numerical data can be found in **S1 Data**.

**
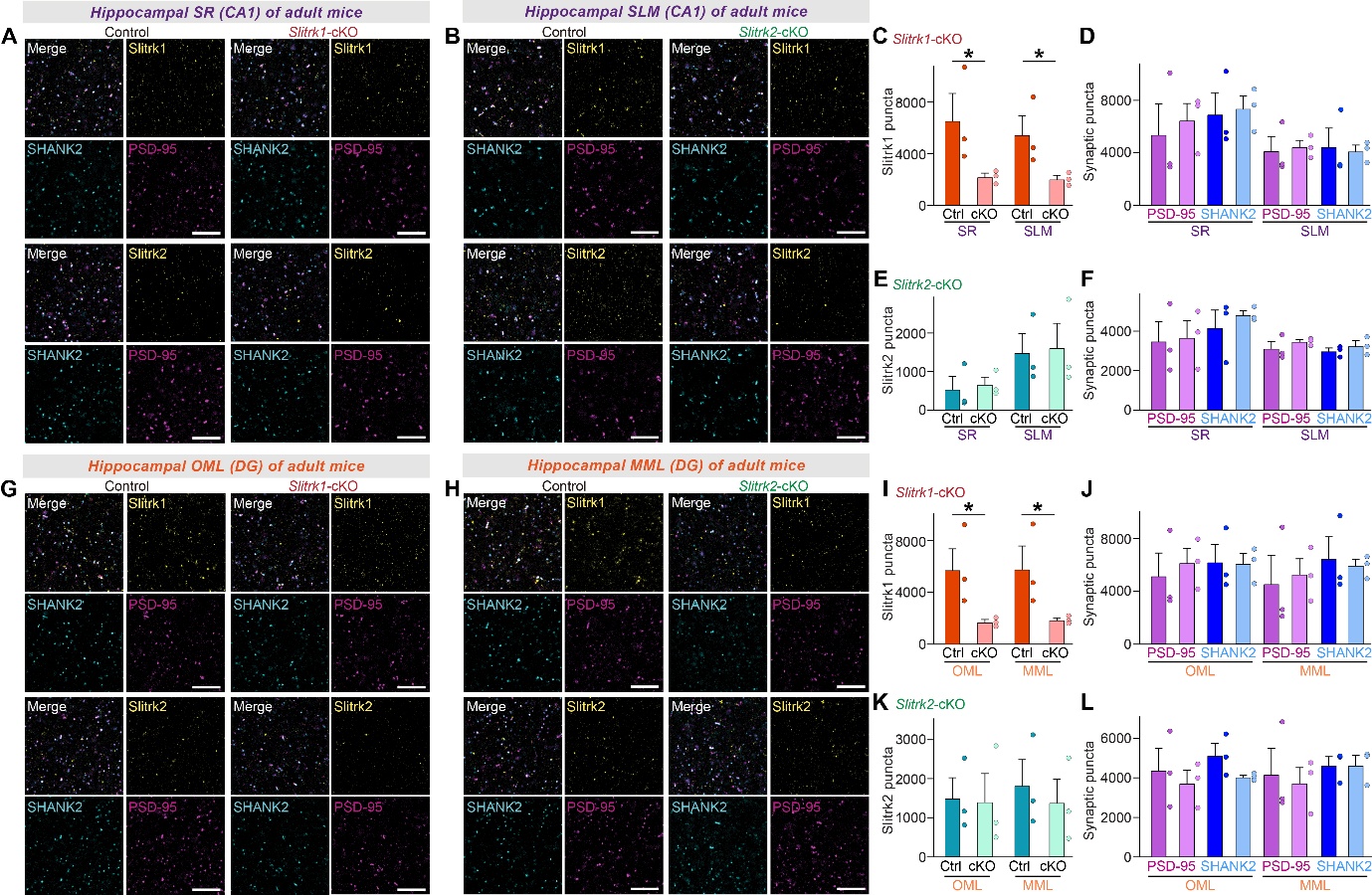
**

**S2 Fig. Validation of Slitrk1 and Slitrk2 antibodies used in eMAP tissues derived from *Slitrk*-cKO mice.**

(**A** and **B**) Immunohistochemical staining-based validation of the antibodies recognizing Slitrk1 (**top**) and Slitrk2 (**bottom**) with the postsynaptic markers, SHANK2 and PSD-95, in the SR (**A**) and SLM (**B**) regions of the CA1 hippocampus of ΔCre control and Cre cKO mice. *Slitrk*-floxed mice were stereotactically injected with AAVs expressing Cre recombinase or inactive Cre (ΔCre; control), and immunohistochemical analyses were performed 3 weeks later. Scale bars = 2 µm (applies to all images). Abbreviations: SR, *stratum radiatum*; SLM, *stratum lacunosum-moleculare*.

(**C** and **D**) Quantitative analysis of (**C**) the total number of Slitrk1 puncta for ΔCre control (Ctrl) and Cre cKO mice in the SR and SLM layers of the CA1 hippocampus and (**D**) the total number of PSD-95 and SHANK2 synaptic puncta from the corresponding SR and SLM layer images analyzed in (**A** and **B**). Data are presented as means ± SEMs (n = 3 mice/group; **p* < 0.05; one-tailed Mann-Whitney *U* test).

(**E** and **F**) Quantitative analysis of (**E**) the total number of Slitrk2 puncta for ΔCre control (Ctrl) and Cre cKO mice in the SR and SLM layers of the CA1 hippocampus and (**F**) the total number of PSD-95 and SHANK2 synaptic puncta from the corresponding SR and SLM layer images analyzed in (**A** and **B**). Data are presented as means ± SEMs (n = 3 mice/group).

(**G** and **H**) Immunohistochemical staining-based validation of antibodies against Slitrk1 (**top**) and Slitrk2 (**bottom**) with postsynaptic markers, SHANK2 and PSD-95 in the OML (**G**) and MML (**H**) regions of the DG of ΔCre control and Cre cKO mice. Scale bars = 2 µm (applies to all images). Abbreviations: OML, outer molecular layer; MML, middle molecular layer.

(**I** and **J**) The same quantitative analysis of Slitrk1, PSD-95, and SHANK2 puncta from (**C** and **D**) but in the OML and MML layers of the DG. Data are presented as means ± SEMs (n = 3 mice/group; **p* < 0.05; one-tailed Mann-Whitney *U* test).

(**K** and **L**) The same quantitative analysis of Slitrk1, PSD-95, and SHANK2 puncta from (**E** and **F**) but in the OML and MML layers of the DG. Puncta were measured from volumetric images from three separate gels (3 images/gel) for each mouse, and values were averaged for each mouse. Data are presented as means ± SEMs (n = 3 mice/group). Numerical data can be found in **S1 Data**.

**
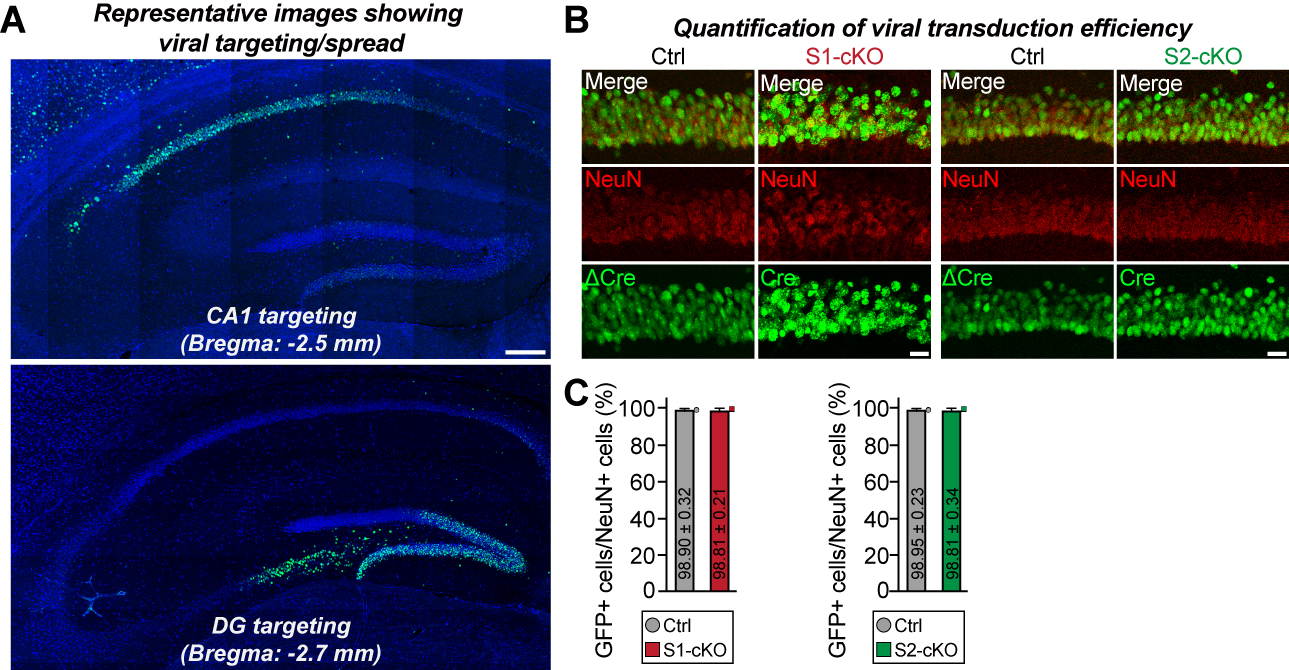
**

**S3 Fig. Immunohistochemical analyses showing the virus targeting and virus transduction efficiency in hippocampal CA1 and DG from adult *Slitrk*-cKO mice.**

(**A**) Representative images showing that stereotactic injections reliably targeted AAVs to the CA1 and dentate gyrus (DG) in adult male *Slitrk*-cKO mice. GFP fluorescence indicates virus-transduced neurons. Scale bar: 200 μm.

(**B**) Immunohistochemical analyses of viral transduction efficiency. Sections were stained with anti-NeuN (to label neurons) and anti-GFP antibodies. Colocalization of GFP with NeuN was used to verify cell-type-specific infection. Scale bar: 50 μm.

(**C**) Quantification of transduction efficiency, expressed as the percentage of GFP⁺/NeuN⁺ double-positive cells among total NeuN⁺ neurons. Data are presented as means ± SEMs (‘n’ denotes number of images; ΔCre, n = 3, Cre, n = 3; two-tailed non-parametric Mann-Whitney *U* test). Numerical data can be found in **S1 Data**.

**S4 Fig.
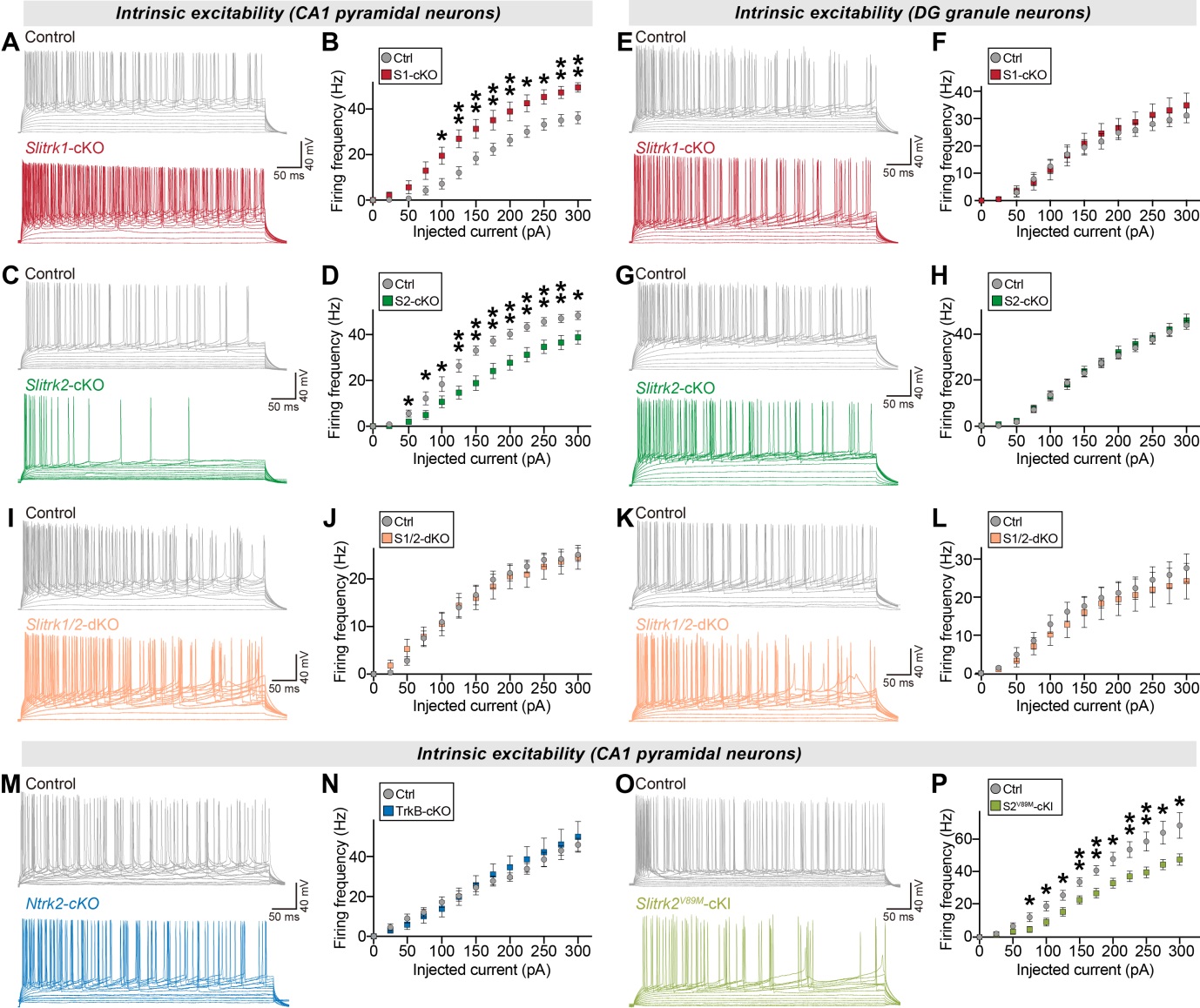
Measurement of cell excitability in hippocampal CA1 pyramidal neurons from *Slitrk1*-cKO, *Slitrk2*-cKO, *Slitrk1/2*-dKO, *Ntrk2*-cKO, and *Slitrk2*^V89M^-cKI mice.**

(**A–D**, **I**, **J**, **M–P**) Whole-cell recordings of action potential (AP) firings from CA1 pyramidal neurons in control (gray), *Slitrk1*-cKO (red), *Slitrk2*-cKO (green), *Slitrk1/2*-cKO (orange), *Ntrk2*-cKO (blue), and *Slitrk2*^V89M^-cKI (light green) hippocampal slices. Representative traces (**A**, **C**, **I**, **M** and **O**) and averages of AP firings (**B**, **D**, **J**, **N** and **P**; control in (**B**), n = 12/3; control in (**D**), n =10/4; control in (**J**), n = 13/4; control in (**N**), n = 14/3; control in (**P**), n = 13/3; *Slitrk1*-cKO, n = 11/4; *Slitrk2*-cKO*,* n = 15/4; *Slitrk1/2*-dKO, n = 11/4; and *Ntrk2*-cKO, n = 15/3; and *Slitrk2*^V89M^-cKI, n = 14/3, where ‘n’ denotes number of cells/mice).

(**E–H**) Same as (**A–D**), except that whole-cell recordings of representative AP firings from DG granule neurons were performed. Representative traces (**E**, **G** and **K**) and averages of AP firings (**F**, H and **L**; control in (**F**), n = 12/4; control in (**H**), n *=* 21/5; control in (**L**), n = 11/4; *Slitrk1*-cKO, n = 12/4; *Slitrk2*-cKO*,* n = 17/5; and *Slitrk1/2*-dKO, n = 10/4). Data are presented as means ± SEMs (**p* < 0.05, ***p* < 0.01; two-tailed non-parametric Mann-Whitney *U* test). Numerical data can be found in **S1 Data**.

**
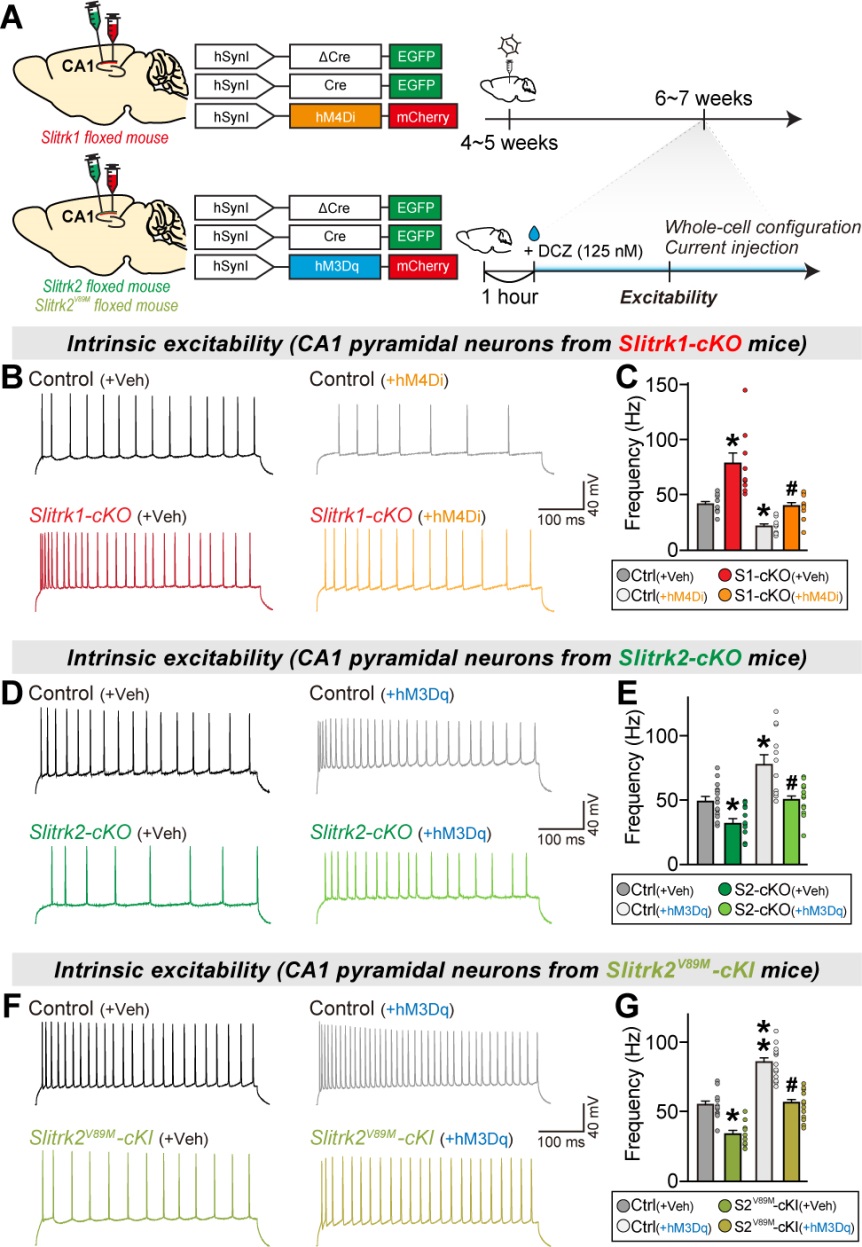
**

**S5 Fig. Validation for the ability of DREADD-based chemogenetics to normalize excitabilities of CA1 pyramidal neurons from adult male *Slitrk*-cKO mice.**

(**A**) Schematic showing AAV injection into the hippocampal CA1 of *Slitrk1*^f/f^, *Slitrk2*^f/f^ and *Slitrk2-*V89M^f/f^ mice. Electrophysiological recordings were performed 2 weeks after injections. (**B** and **C**) Whole-cell recordings of action potential (AP) firings from CA1 pyramidal neurons in control (dark-grey), *Slitrk1*-cKO (red), control+hM4Di (light-grey), and *Slitrk1*-cKO+hM4Di (orange) hippocampal slices. Representative traces (**B**) and averages of AP firings (**C**; control, n = 10/2; *Slitrk1*-cKO, n = 9/2; control+hM4Di, n = 10/2; *Slitrk1*-cKO+hM4Di, n = 10/2, where n denotes the number of cells/mice). (**D** and **E)** Whole-cell recordings of AP firings from CA1 pyramidal neurons in control (dark-grey), *Slitrk2*-cKO (green), control+hM4Di (light-grey), and *Slitrk2*-cKO+hM4Di (light-green) hippocampal slices. Representative traces (**D**) and averages of AP firings (**E**; control, n = 16/2; *Slitrk2*-cKO, n = 13/2; control+hM4Dq, n = 12/2; *Slitrk2*-cKO+hM3Dq, n = 14/2, where n denotes the number of cells/mice). (**F** and **G**) Whole-cell recordings of AP firings from CA1 pyramidal neurons in control (dark-grey), *Slitrk2^V89M^*-cKI (lime), control+hM4Di (light-grey), and *Slitrk2^V89M^*-cKI +hM4Di (dark-lime) hippocampal slices. Representative traces (**F**) and averages of AP firings (**G**; control, n = 11/2; *Slitrk2^V89M^*-cKI, n = 12/2; control+hM3Dq, n = 13/2; *Slitrk2^V89M^*-cKI+hM3Dq, n = 14/2, where n denotes the number of cells/mice). Data are presented as means ± SEMs (**p* < 0.05, ***p* < 0.01, ^#^ < 0.05; ‘#’ indicates statistical comparisons with their counterparts; nonparametric Kruskal-Wallis test with Dunn’s *post hoc* test). Numerical data can be found in **S1 Data**.

**
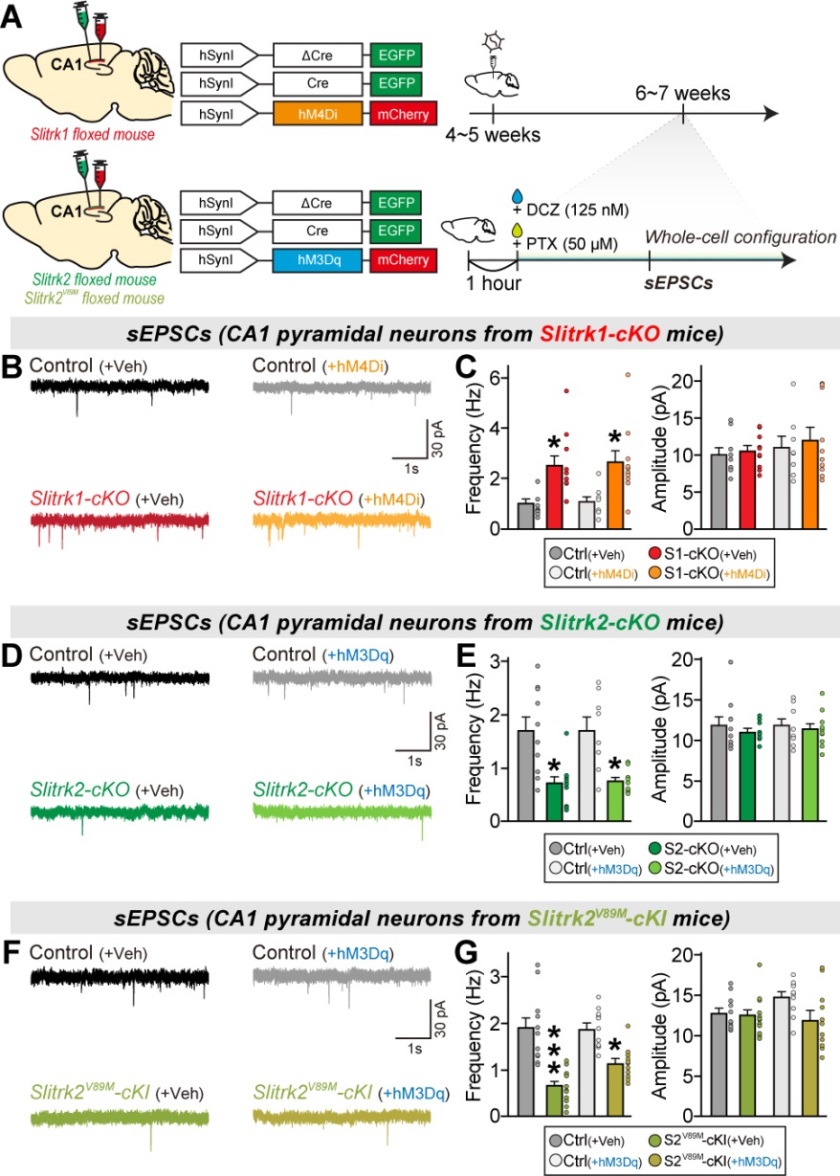
**

**S6 Fig. Normalizing excitability of CA1 pyramidal neurons from adult male *Slitrk*-cKO and *Slitrk2*^V89M^-cKI mice does not affect altered spontaneous excitatory synaptic transmission.**

(**A**) Schematic showing AAV injection into the hippocampal CA1 of *Slitrk1*^f/f^, *Slitrk2*^f/f^ and *Slitrk2-*V89M^f/f^ mice. Electrophysiological recordings were performed 2 weeks after injections. (**B** and **C**) Whole-cell recordings of sEPSCs from CA1 pyramidal neurons in control (dark-grey), *Slitrk1*-cKO (red), control+hM4Di (light-grey), *Slitrk1*-cKO+hM4Di (orange) hippocampal slices. Representative traces (**B**) and averages of AP firings (**C**; control, n = 9/2; *Slitrk1*-cKO, n = 10/2; control+hM4Di, n = 8/2; *Slitrk1*-cKO+hM4Di, n = 10/2, where n denotes the number of cells/mice). (**D** and **E)** Whole-cell recordings of sEPSCs from CA1 pyramidal neurons in control (dark-grey), *Slitrk2*-cKO (green), control+hM4Di (light-grey), *Slitrk2*-cKO+hM4Di (light-green) hippocampal slices. Representative traces (**D**) and averages of AP firings (**E**; control, n = 10/2; *Slitrk2*-cKO, n = 10/2; control+hM4Dq, n = 8/2; *Slitrk2*-cKO+hM3Dq, n = 10/2, where n denotes the number of cells/mice). (**F** and **G**) Whole-cell recordings of sEPSCs from CA1 pyramidal neurons in control (dark-grey), *Slitrk2^V89M^*-cKI (lime), control+hM4Di (light-grey), *Slitrk2^V89M^*-cKI +hM4Di (dark-lime) hippocampal slices. Representative traces (**F**) and averages of AP firings (**G**; control, n = 11/2; *Slitrk2^V89M^*-cKI, n = 12/2; control+hM4Dq, n = 11/2; *Slitrk2^V89M^*-cKI+hM3Dq, n = 11/2, where n denotes the number of cells/mice). Data are presented as means ± SEMs (**p* < 0.05, ****p* < 0.001; nonparametric Kruskal-Wallis test with Dunn’s *post hoc* test). Numerical data can be found in **S1 Data**.

**
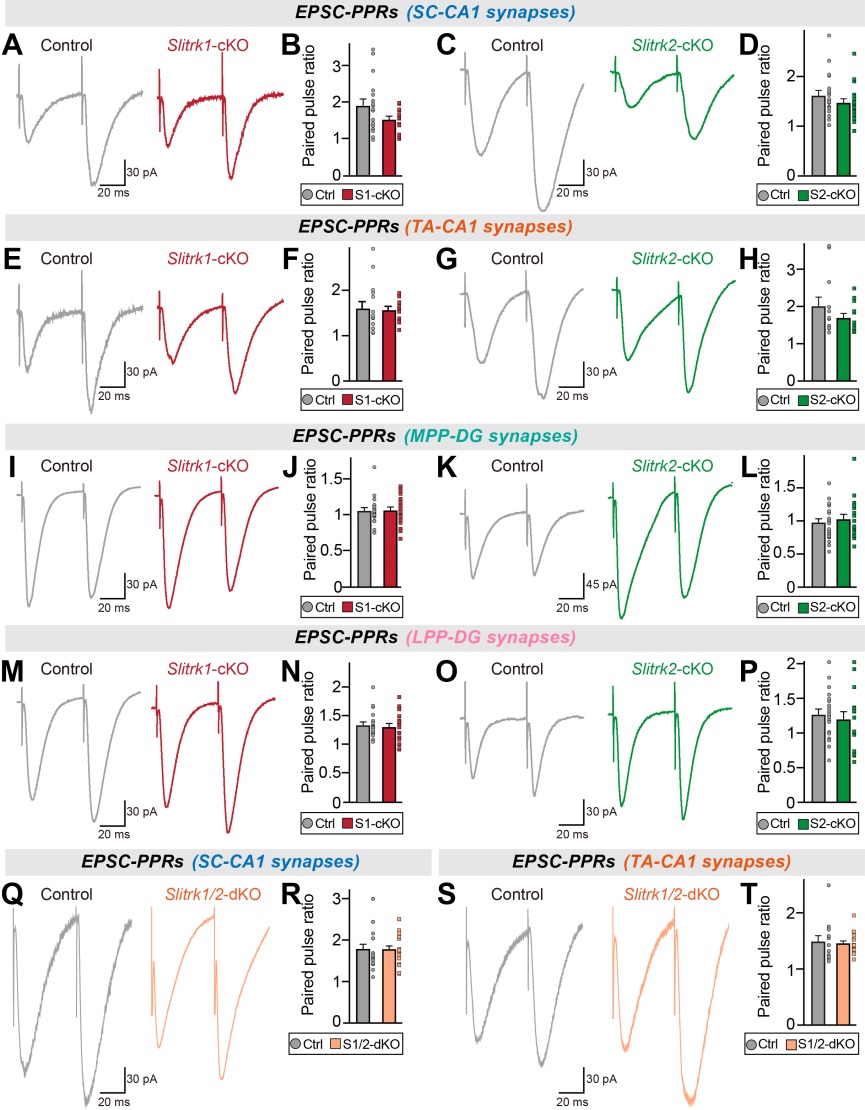
**

**S7 Fig. Measurement of paired pulse ratio in hippocampal CA1 pyramidal neurons and DG granule neurons from *Slitrk1*-cKO, *Slitrk2*-cKO and *Slitrk1/2*-dKO mice.**

(**A–D**) Whole-cell recordings of eEPSC-PPRs at SC-CA1 synapses in control (gray), *Slitrk1*-cKO (red), and *Slitrk2*-cKO (green) hippocampal slices. Representative traces (**A** and **C**) and averages of eEPSC-PPRs (**B** and **D**; control in (**B**), n = 17/5; control in (**D**), n = 19/5; *Slitrk1*-cKO, n = 13/4; *Slitrk2*-cKO*,* n = 20/5, where n denotes the number of cells/mice). (**E–H**) Same as (**A–D**), except that whole-cell recordings of eEPSCs at TA-CA1 synapses were performed. Representative traces (**E** and **G**) and averages of eEPSC-PPRs (**F** and **H**; control in (**F**), n = 15/5; control in (**H**), n = 12/4; *Slitrk1*-cKO, n = 12/4; *Slitrk2*-cKO*,* n = 12/4). (**I–L**) Same as (**A–D**), except that whole-cell recordings of eEPSC-PPRs at MPP-DG synapses were performed. Representative traces (**I** and **K**) and averages of eEPSC-PPRs (**J** and **L**; n denotes the number of cells/mice; control in (**J**), n = 20/5; control in (**L**), n = 23/5; *Slitrk1*-cKO, n = 21/5; *Slitrk2*-cKO*,* n = 19/5). (**M–P**) Same as (**A–D**), except that whole-cell recordings of eEPSC-PPRs at LPP-DG synapses were performed. Representative traces (**M** and **O**) and averages of eEPSC-PPRs (**N** and **P**; control in (**N**), n = 20/5; control in (**P**), n = 20/5; *Slitrk1*-cKO, n = 21/5; *Slitrk2*-cKO*,* n = 16/5). Data are presented as means ± SEMs (two-tailed non-parametric Mann-Whitney *U* test). (**Q** and **R**) Whole-cell recordings of eEPSC-PPRs at SC-CA1 synapses of control (gray) and *Slitrk1/2*-dKO (light orange) mice. Representative traces (**Q**) and averages of eEPSC-PPRs (**R**; control, n = 13/4; *Slitrk1/2*-dKO*,* n = 15/3). Data are presented as means ± SEMs (two-tailed non-parametric Mann-Whitney *U* test). (**S** and **T**) Same as (**Q** and **R**), except that whole-cell recordings of eEPSC-PPRs at TA-CA1 synapses were performed. Representative traces (**S**) and averages of eEPSC-PPRs (**T**; control, n = 12/3; *Slitrk1/2*-dKO, n = 15/3). Data are presented as means ± SEMs (two-tailed non-parametric Mann-Whitney *U* test). Numerical data can be found in **S1 Data**.


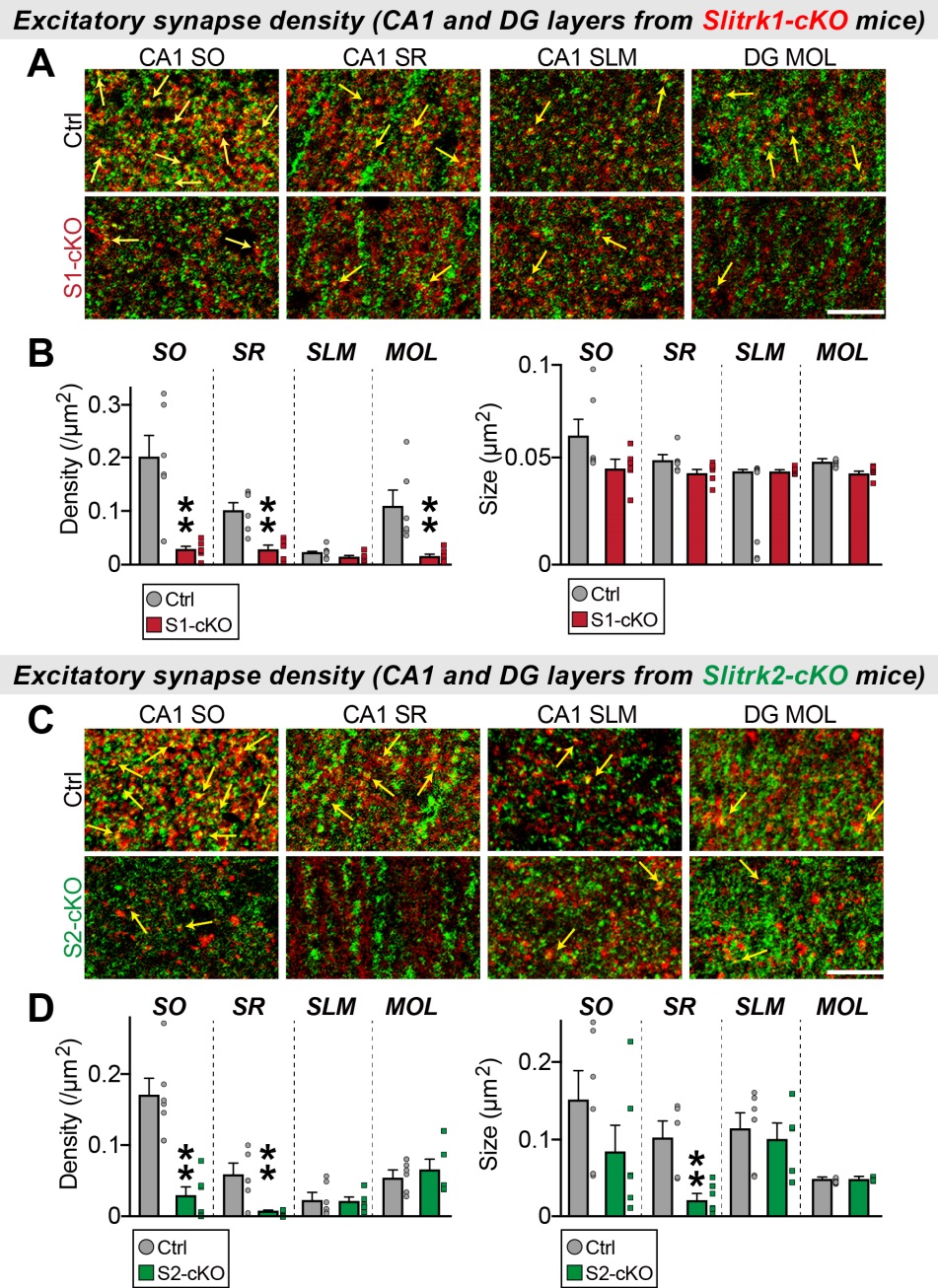


**S****8 Fig. Immunohistochemical analyses showing measured changes in excitatory synapse density of hippocampal CA1 and DG from adult *Slitrk*-cKO mice.**

(**A** and **B**) Representative images (**A**) and summary graphs (**B**) of immunohistochemistry results for excitatory synapses in the hippocampal CA1 and DG layers of adult male control (ΔCre) and *Slitrk1*-cKO (Cre) mice. Scale bar: 20 μm. Data are presented as means ± SEMs (‘n’ denotes number of mice; SO: ΔCre, n = 6, Cre, n = 6; SP: ΔCre, n = 6, Cre, n = 5; SR: ΔCre, n = 6, Cre, n = 6; SLM: ΔCre, n = 6, Cre, n = 6; ***p* < 0.01; two-tailed non-parametric Mann-Whitney *U* test). Numerical data can be found in **S1 Data**.

**
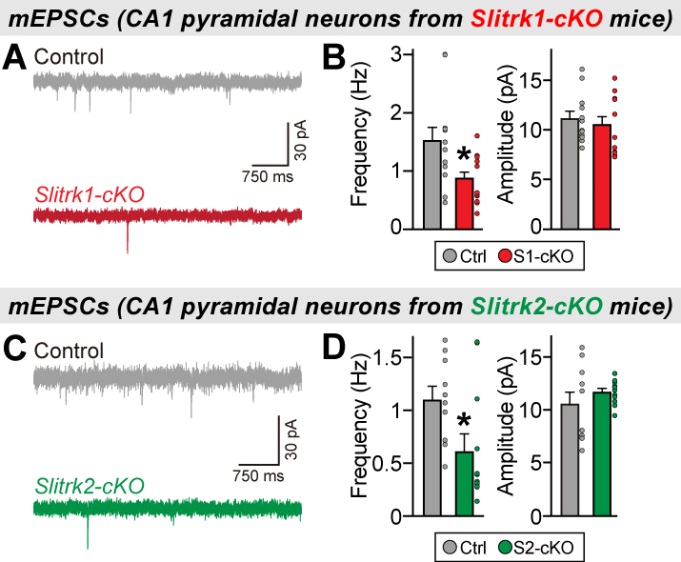
**

**S9 Fig. Measurement of miniature excitatory postsynaptic currents in hippocampal CA1 pyramidal neurons from adult male *Slitrk1*-cKO mice.**

(**A** and **B**) Whole-cell recordings of mEPSCs from CA1 pyramidal neurons in control (gray), *Slitrk1*-cKO (red) hippocampal slices. Representative traces (A) and averages of mEPSCs (B; control, n = 12/2; *Slitrk1*-cKO, n = 11/2, where ‘n’ denotes number of cells/mice).

(**C** and **D**) Whole-cell recordings of mEPSCs from CA1 pyramidal neurons in control (gray), *Slitrk2*-cKO (green) hippocampal slices. Representative traces (**C**) and averages of mEPSCs (**D**; control, n = 12/3; *Slitrk2*-cKO, n = 10/3, where ‘n’ denotes number of cells/mice). Data are presented as means ± SEMs (**p* < 0.05; two-tailed non-parametric Mann-Whitney *U*test). Numerical data can be found in **S1 Data**.

**
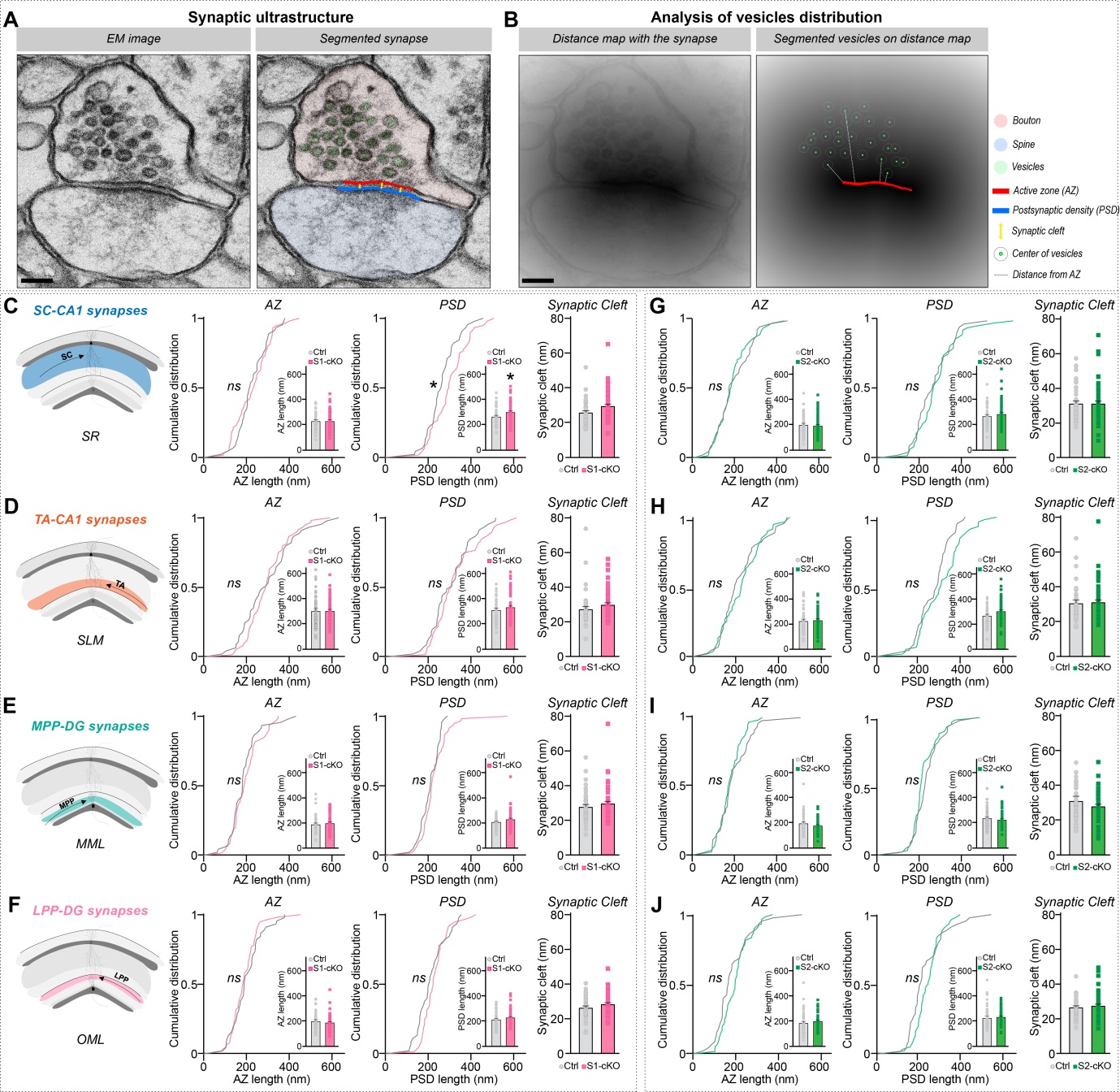
S10 Fig. Deletion of Slitrk1 or Slitrk2 does not alter the ultrastructure of**

**synapses at hippocampal CA1 and DG circuitries.**

(**A**) Representative electron micrographic images (**left**) used for ultrastructure analysis of synapses at high magnification. Each component within the synapse was segmented (**right**). Scale bars, 100 nm.

(**B**) Distance map containing information (**left**) about the distance from an active zone (AZ). Vesicle locations marked on the distance map (**right**). Scale bars, 100 nm (applies to all images).

(**C–F**) Quantification of AZ length (**left**; cumulative distribution plot and bar graph), length of postsynaptic density (PSD) (**middle**; cumulative distribution plot and bar graph), and width of synaptic cleft (**right**) at the SC-CA1 (**C**), TA-CA1 (**D**), MPP-DG (**E**) and LPP-DG (**F**) synapses of *Slitrk1*-cKO compared with control mice.

(**G–J**) Quantification of AZ length (**left**), PSD length (**middle**), and width of synaptic cleft (**right**) at the SC-CA1 (**G**), TA-CA1 (**H**), MPP-DG (**I**) and LPP-DG (**J**) synapses of *Slitrk2*-cKO compared with control mice. Data represents means ± SEMs (control, n = 45/3; *Slitrk1*-cKO or *Slitrk2*-cKO, n = 60/4, where n denotes the number of synapses/mice; **p*< 0.05; n.s., not significant; Kolmogorov-Smirnov test). Numerical data can be found in **S1 Data**.

**
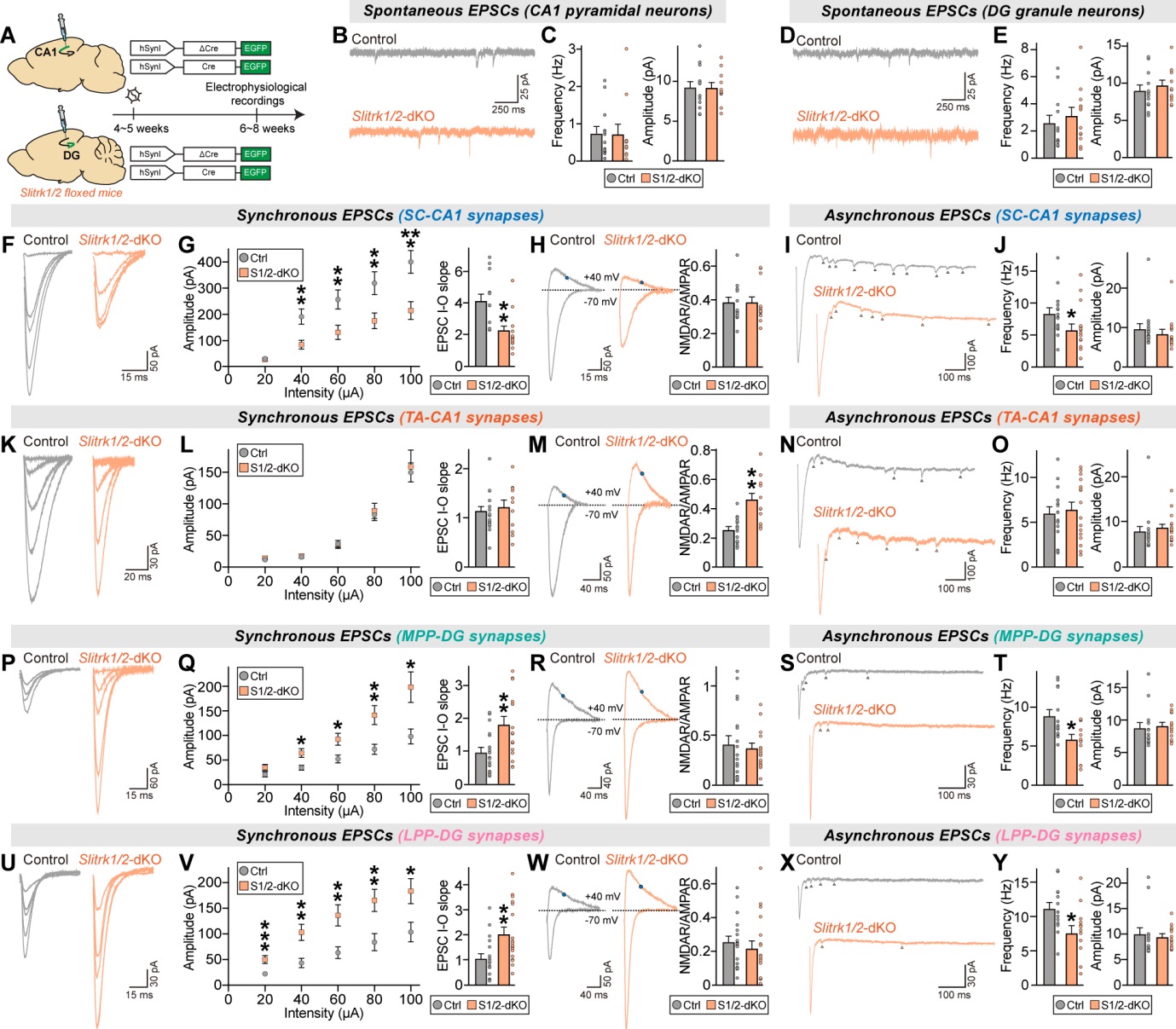
**

**S11 Fig. Simultaneous deletion of Slitrk1 and Slitrk2 produces phenotypes induced by ablating each Slitrk paralog from microcircuits of the hippocampal CA1 and DG regions.**

(**A**) Schematic showing AAV injections into the hippocampal CA1 or DG of *Slitrk1/2*^f/f^ mice. Electrophysiological recordings were performed 2 weeks after injections.

(**B** and **C**) Whole-cell recordings of sEPSCs from CA1 pyramidal neurons of control (gray) and *Slitrk1/2*-dKO (light orange) hippocampal slices. Representative traces (**B**) and averages of eEPSCs (**C**; control, n = 13/3; *Slitrk1/2*-dKO, n = 11/3, where ‘n’ denotes number of cells/mice).

(**D** and **E**) Whole-cell recordings of sEPSCs from DG granule neurons of control and *Slitrk1/2*-dKO hippocampal slices. Representative traces (**D**) and averages of eEPSCs (**E**; control, n = 12/3; *Slitrk1/2*-dKO, n = 12/3).

(**F** and **G**) Whole-cell recordings of eEPSCs at SC-CA1 synapses of control and *Slitrk1/2*-dKO hippocampal slices. Representative traces (**F**) and averages of eEPSCs (**G**; control, n = 12/3; *Slitrk1/2*-dKO, n = 12/3).

(**H**) Representative traces of NMDAR/AMPAR-EPSCs at SC-CA1 synapses of control and *Slitrk1/2*-dKO hippocampal slices (**left**) and averages of NMDAR/AMPAR-EPSCs (**right**; control, n = 12/3; *Slitrk1/2*-dKO, n = 12/2).

(**I** and **J**) Whole-cell recordings of aEPSCs at SC-CA1 synapses of control and *Slitrk1/2*-dKO hippocampal slices. Representative traces (**I**) and averages of aEPSCs (**J**; control, n = 14/5; *Slitrk1/2*-dKO, n = 13/5).

(**K** and **L**) Representative traces of eEPSCs at TA-CA1 synapses of control and *Slitrk1/2*-dKO hippocampal slices (**K**) and averages of NMDAR/AMPAR-EPSCs (**L**; control, n = 15/3; *Slitrk1/2*-dKO, n = 11/3).

(**M**) Representative traces of NMDAR/AMPAR-EPSCs at TA-CA1 synapses of control and *Slitrk1/2*-dKO hippocampal slices (**left**) and averages of NMDAR/AMPAR-EPSCs (**right**; control, n = 15/3; *Slitrk1/2*-dKO, n = 11/3).

(**N** and **O**) Whole-cell recordings of aEPSCs at TA-CA1 synapses of control and *Slitrk1/2*-dKO hippocampal slice. Representative traces (**N**) and averages of aEPSCs (**O**; control, n = 14/5; *Slitrk1/2*-dKO, n = 13/5).

(**P** and **Q**) Whole-cell recordings of eEPSCs at MPP-DG synapses of control and *Slitrk1/2*-dKO hippocampal slices. Representative traces (**P**) and averages of eEPSCs (**Q**; control, n = 18/5; *Slitrk1/2*-dKO, n = 18/5).

(**R**) Representative traces of NMDAR/AMPAR-EPSCs at MPP-DG synapses of control and *Slitrk1/2*-dKO hippocampal slices (**left**) and averages of NMDAR/AMPAR-EPSCs (**right**; control, n = 18/5; *Slitrk1/2*-dKO, n = 18/5).

(**S** and **T**) Whole-cell recordings of aEPSCs at MPP-DG synapses of control and *Slitrk1/2*-dKO hippocampal slices. Representative traces (**S**) and averages of aEPSCs (**T**; control, n = 14/5; *Slitrk1/2*-dKO, n = 13/5).

(**U** and **V**) Representative traces of eEPSCs at LPP-DG synapses of control and *Slitrk1/2*-dKO hippocampal slices (**U**) and averages of NMDAR/AMPAR-EPSCs (**V**; control, n = 18/5; *Slitrk1/2*-dKO, n = 18/5).

(**W**) Representative traces of NMDAR/AMPAR-EPSCs at LPP-DG synapses of control and *Slitrk1/2*-dKO hippocampal slices (**left**) and averages of NMDAR/AMPAR-EPSCs (**right**; control, n = 18/5; *Slitrk1/2*-dKO, n = 18/5).

(**X** and **Y**) Whole-cell recordings of aEPSCs at LPP-DG synapses of control and *Slitrk1/2*-dKO hippocampal slice. Representative traces (**X**) and averages of aEPSCs (**Y**; control, n = 14/5; *Slitrk1/2*-dKO, n = 13/5). Data are presented as means ± SEMs (**p* < 0.05, ***p* < 0.01, ****p* < 0.001; two-tailed non-parametric Mann-Whitney *U* test). Numerical data can be found in **S1 Data**.

**
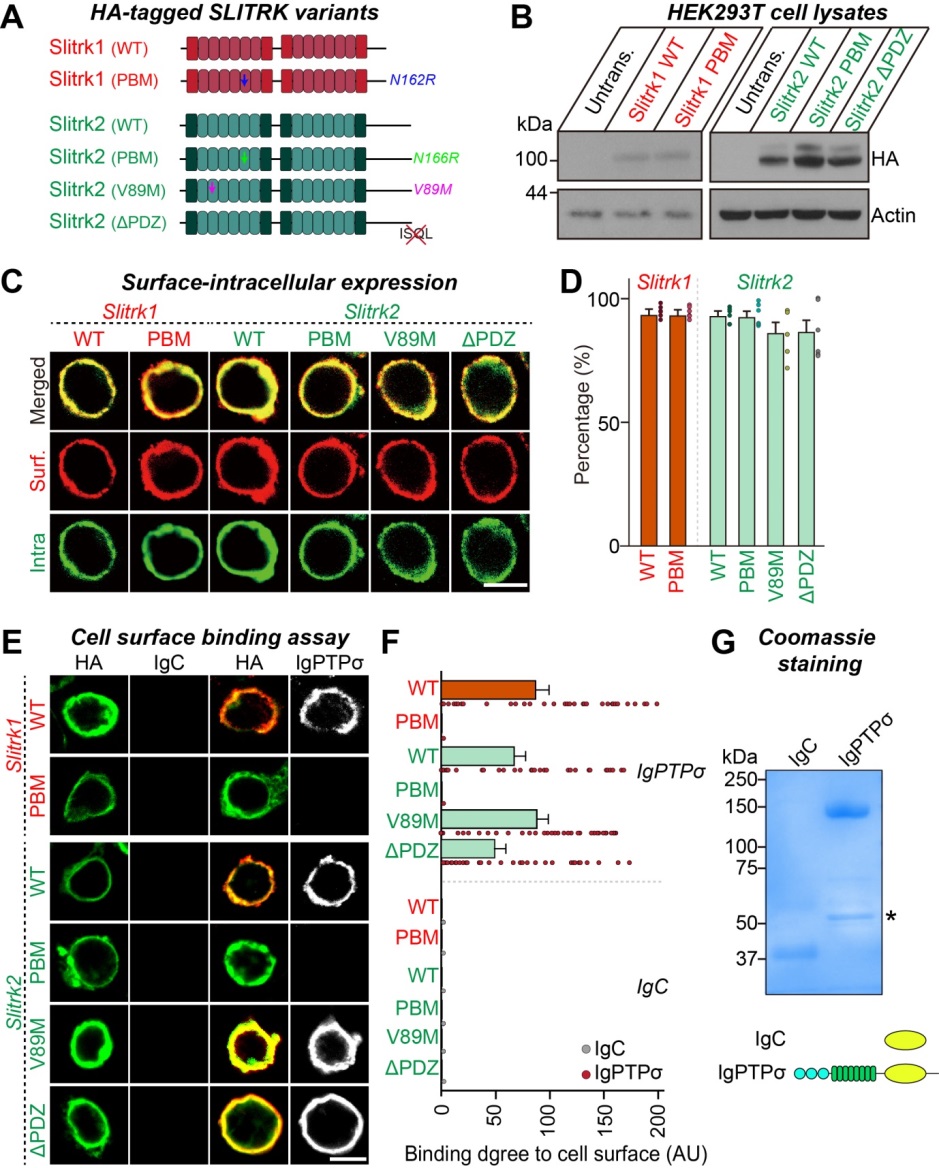
**

**S12 Fig. Characterization of SLITRK expression vectors used in the current study.**

(**A**) Diagrams illustrating HA-tagged SLITRK variants (SLITRK1 WT, SLITRK1 PBM, SLITRK2 WT, SLITRK2 PBM, SLITRK2 V89M and SLITRK2 ΔPDZ) used in the molecular replacement experiments.

(**B**) Immunoblot analyses monitoring the expression levels of the indicated SLITRK1 (**left**) and SLITRK2 (**right**) variants in HEK293T cells.

(**C**) Representative images of transfected HEK293T cells showing the expression and surface transport activity of SLITRK variants. Transfected cells were fixed (not permeabilized) and incubated with anti-HA antibody to detect the extracellular region of SLITRK (red). The intracellular level of SLITRK variants was determined by incubation with an anti-HA antibody (green) after permeabilization. Surf., surface; Intra., intracellular. Scale bar, 10 µm (applies to all images).

(**D**) Quantification of surface transport activity of SLITRK variants. Data are presented as means ± SEMs (n = 6 images/group; nonparametric Kruskal-Wallis test with Dunn’s *post hoc* test).

(**E** and **F**) Cell surface-binding assays. Representative images (**E**) and summary graphs (**F**) showing HEK293T cells expressing N-terminally HA-tagged SLITRK variants incubated with purified Ig-fused PTPσ (Ig-PTPσ) or IgC alone (control) and analyzed by immunofluorescence imaging for Ig-fusion proteins (red) and HA (green). Data are presented as means ± SEMs (n = 38–76 cells/group). Scale bar, 10 µm (applies to all images).

(**G**) Coomassie-stained gel of recombinant IgC and IgPTPσ used for cell-surface binding assays. The band denoted by an asterisk is likely a degradation product of the full-length PTPσ Ig-fusion proteins. Numerical data can be found in **S1 Data**.

**
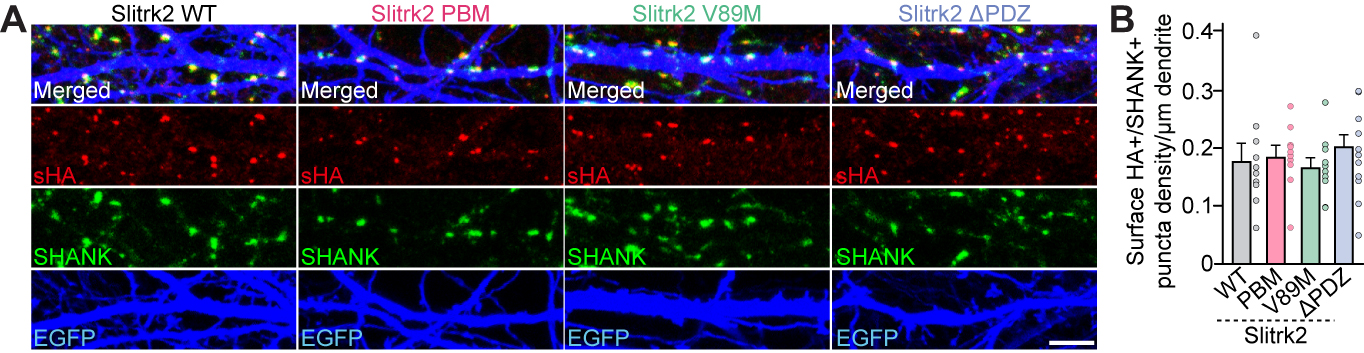
**

**S13 Fig Slitrk2 variants used in the current study exhibit comparable surface expression in the excitatory synapses of cultured hippocampal neurons.**

(**A**) Representative images showing the distribution of HA-tagged Slitrk2 WT and its variants (Slitrk2 PBM, Slitrk2 V89M and Slitrk2 ΔPDZ) in cultured hippocampal neurons. Neurons were transfected at DIV7 and analyzed at DIV14 by immunofluorescence staining using anti-EGFP (blue), anti-HA (red), and anti-SHANK (green) antibodies. Scale bar, 10 µm (applies to all images). (**B**) Density quantification of HA^+^SHANK^+^ puncta. Data are presented as means ± SEMs (n = 10 cells per group). Abbreviation: sHA, surface HA. Numerical data can be found in **S1 Data**.

**
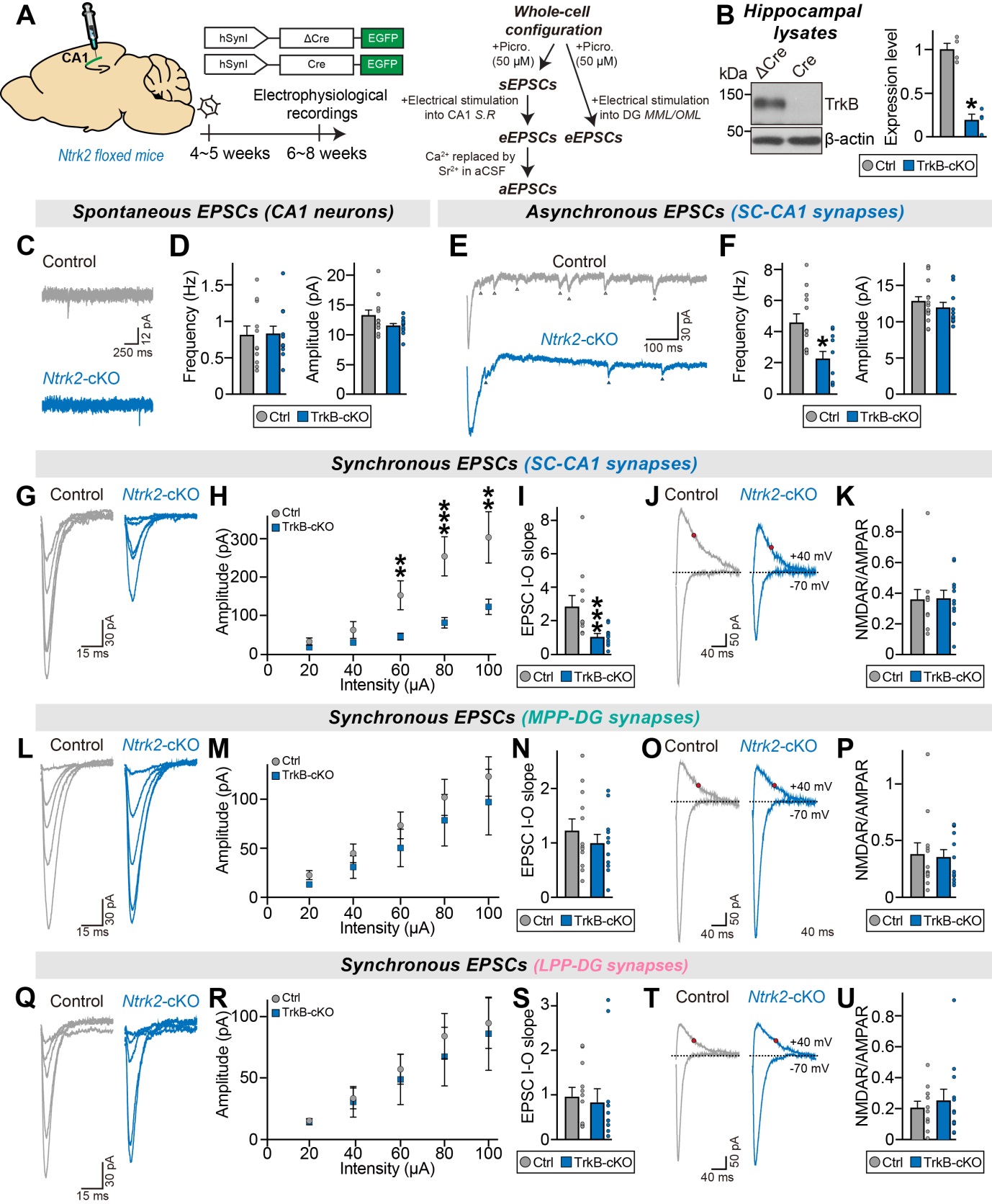
S14 Fig. TrkB is required to regulate evoked synchronous and asynchronous synaptic transmission in Schaffer collateral excitatory synapses of hippocampal CA1 pyramidal neurons.**

(**A**) Schematic showing injections of the indicated AAVs into the hippocampal CA1 or DG of *Ntrk2*^f/f^ mice. Electrophysiological recordings were performed 2 weeks after injections to monitor alterations in spontaneous, synchronous and asynchronous synaptic transmission.

(**B**) Representative immunoblotting image and quantitative analyses of the level of TrkB protein in hippocampal lysates infected with AAVs expressing Cre recombinase. Data are presented as means ± SEMs (n = 4 independent experiments; **p* < 0.05; two-tailed non-parametric Mann-Whitney *U* test).

(**C** and **D**) Whole-cell recordings of sEPSCs from CA1 pyramidal neurons of control (gray) and *Ntrk2*-cKO (blue) hippocampal slices. Representative traces (**C**) averages of sEPSCs (**D**; control, n = 11/2; *Ntrk2*-cKO, n = 11/2, where ‘n’ denotes the number of cells/mice).

(**E** and **F**) Whole-cell recordings of aEPSCs at SC-CA1 synapses of control and *Ntrk2*-cKO hippocampal slices. Representative traces (**E**) and averages of aEPSCs (**F**; control, n = 13/3; *Ntrk2*-cKO, n = 10/3).

(**G–K**) Whole-cell recordings of eEPSCs at SC-CA1 synapses of control and *Ntrk2*-cKO hippocampal slices. Representative traces (**G**) and averages of eEPSCs (**H** and **I**; control, n = 11/3; *Ntrk2*-cKO, n = 11/3). Representative traces of NMDAR/AMPAR-EPSC at SC-CA1 synapses of control and *Ntrk2*-cKO hippocampal slices (**J**) and averages of NMDAR/AMPAR-EPSCs (**K**; control, n = 11/3; *Ntrk2*-cKO, n = 11/3).

(**L–P**) Whole-cell recordings of eEPSCs at MPP-DG synapses of control and *Ntrk2*-cKO hippocampal slices. Representative traces (**L**) and averages of eEPSCs (**M** and **N**; control, n = 12/4; *Ntrk2*-cKO, n = 12/4). Representative traces of NMDAR/AMPAR-EPSCs at MPP-DG synapses of control and *Ntrk2*-cKO hippocampal slices (**O**) and averages of NMDAR/AMPAR-EPSCs (**P**; control, n = 12/4; *Ntrk2*-cKO, n = 12/4).

(**Q–U**) Whole-cell recordings of eEPSCs at LPP-DG synapses of control and *Ntrk2*-cKO hippocampal slices. Representative traces (**Q**) and averages of eEPSCs (**R** and **S**; control, n = 12/4; *Ntrk2*-cKO, n = 12/4). Representative traces of NMDAR/AMPAR-EPSC at LPP-DG synapses of control and *Ntrk2*-cKO hippocampal slices (**T**) and averages of NMDAR/AMPAR-EPSCs (**U**; control, n = 12/4; *Ntrk2*-cKO, n = 12/4). Data are presented as means ± SEMs (**p* < 0.05, ***p* < 0.01, ****p* < 0.001; two-tailed non-parametric Mann-Whitney *U* test). Numerical data can be found in **S1 Data**.

**
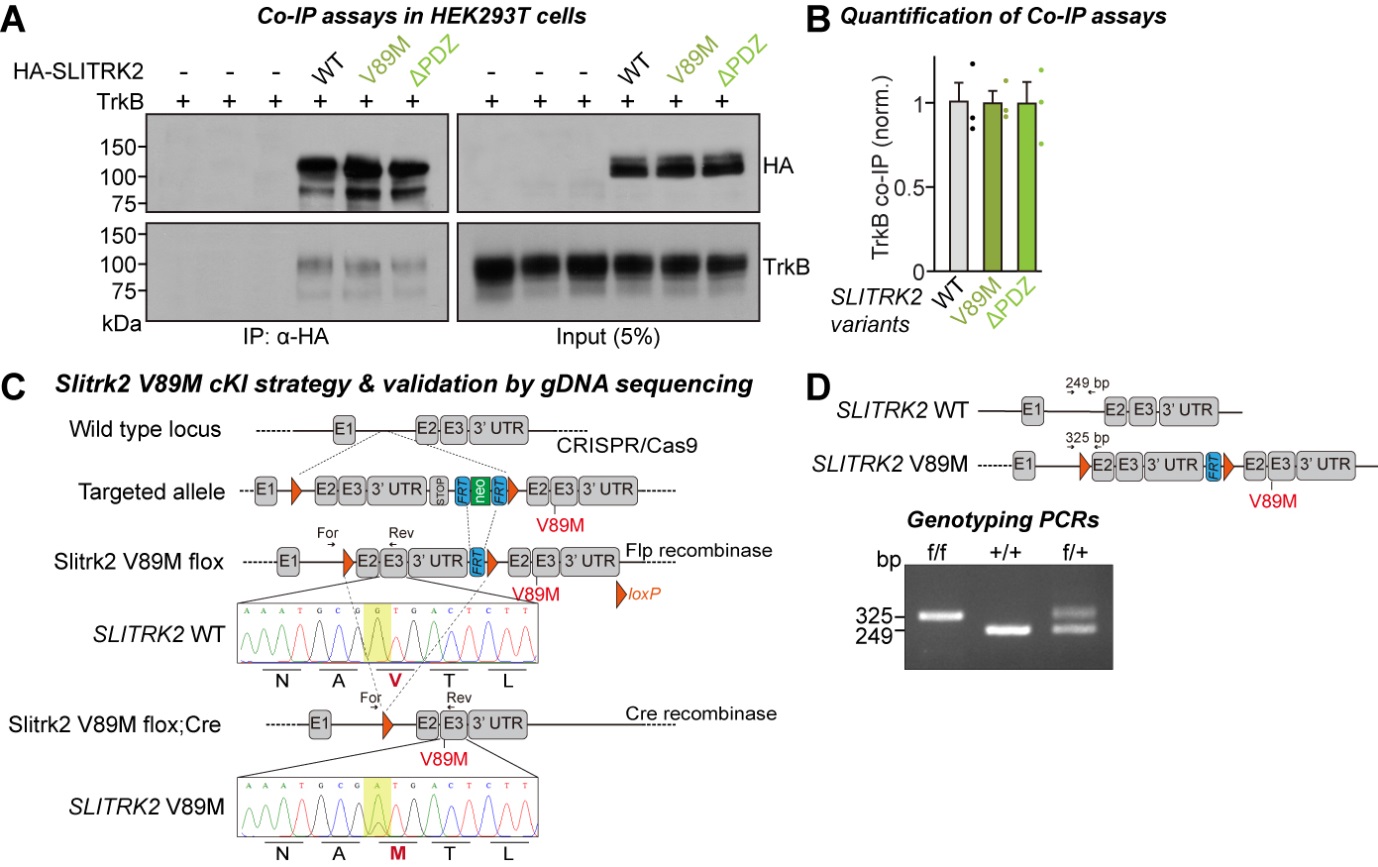
**

**S15 Fig. Substituting valine for methionine at residue 89 or deleting the C-terminal PDZ domain-binding sequence in SLITRK2 does not affect its interaction with TrkB.**

(**A** and **B**) Coimmunoprecipitation experiment showing that SLITRK2 V89M and SLITRK2 ΔPDZ each interact with TrkB, comparable to SLITRK2 WT. HEK293T cells were transfected with HA-tagged SLITRK2 variant alone or together with untagged TrkB construct, followed by coimmunoprecipitation of SLITRK2 with TrkB. (A) Representative immunoblot visualized by ECL; and (**B**) quantitative bar graphs of coimmunoprecipitation efficiency. Data are presented as means ± SEMs (n = 3 independent experiments; nonparametric Kruskal-Wallis test with Dunn’s *post hoc* test). Input, 5%.

(**C**) Knockin strategy used to generate *Slitrk2*^V89M^-cKI mice and its validation. One LoxP site each was inserted upstream of exon 2 (E2) and downstream of the 3’UTR of the murine *Slitrk2* gene and the p.V89M mutation was introduced into exon 3 (E3) by overlap extension PCR. Black arrows indicate forward and reverse primers used for genotyping. Genomic DNA was prepared from hippocampal CA1 lysates injected with AAV-Cre or AAV-ΔCre, followed by PCR amplification using the indicated primers (F, forward; R, reverse). Cre recombinase deleted E2 and E3, causing constitutive expression of *Slitrk2* p.V89M mRNA.

(**D**) PCR genotyping of *Slitrk2*^V89M^-floxed mice. The band size for the *Slitrk2*^V89M^-floxed allele was 249 bp. Numerical data can be found in **S1 Data**.

**
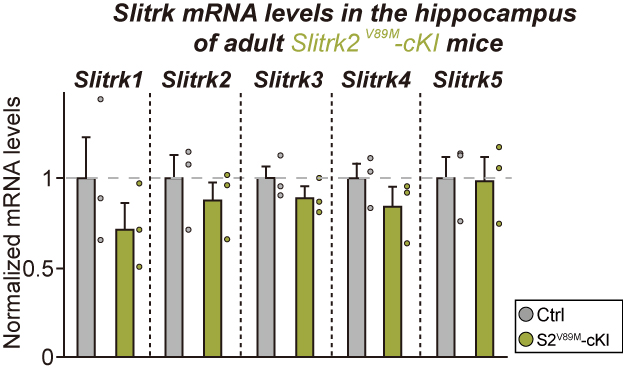
**

**S16 Fig. Measurement of *Slitrk* mRNA levels in adult male *Slitrk2*^V89M^-cKI mice.**

Quantitative RT-PCR analyses measuring mRNA levels of Slitrk paralogs in hippocampal tissues from adult male *Slitrk2*^V89M^-cKI mice. qRT-PCRs were performed using Slitrk paralog-specific probes. Gene expression levels were normalized with respect to a housekeeping gene (GAPDH). Data are presented as means ± SEMs (‘n’ denote number of mice; ΔCre, n = 3; Cre, n = 3; two-tailed non-parametric Mann-Whitney *U* test). Numerical data can be found in **S1 Data**.

**
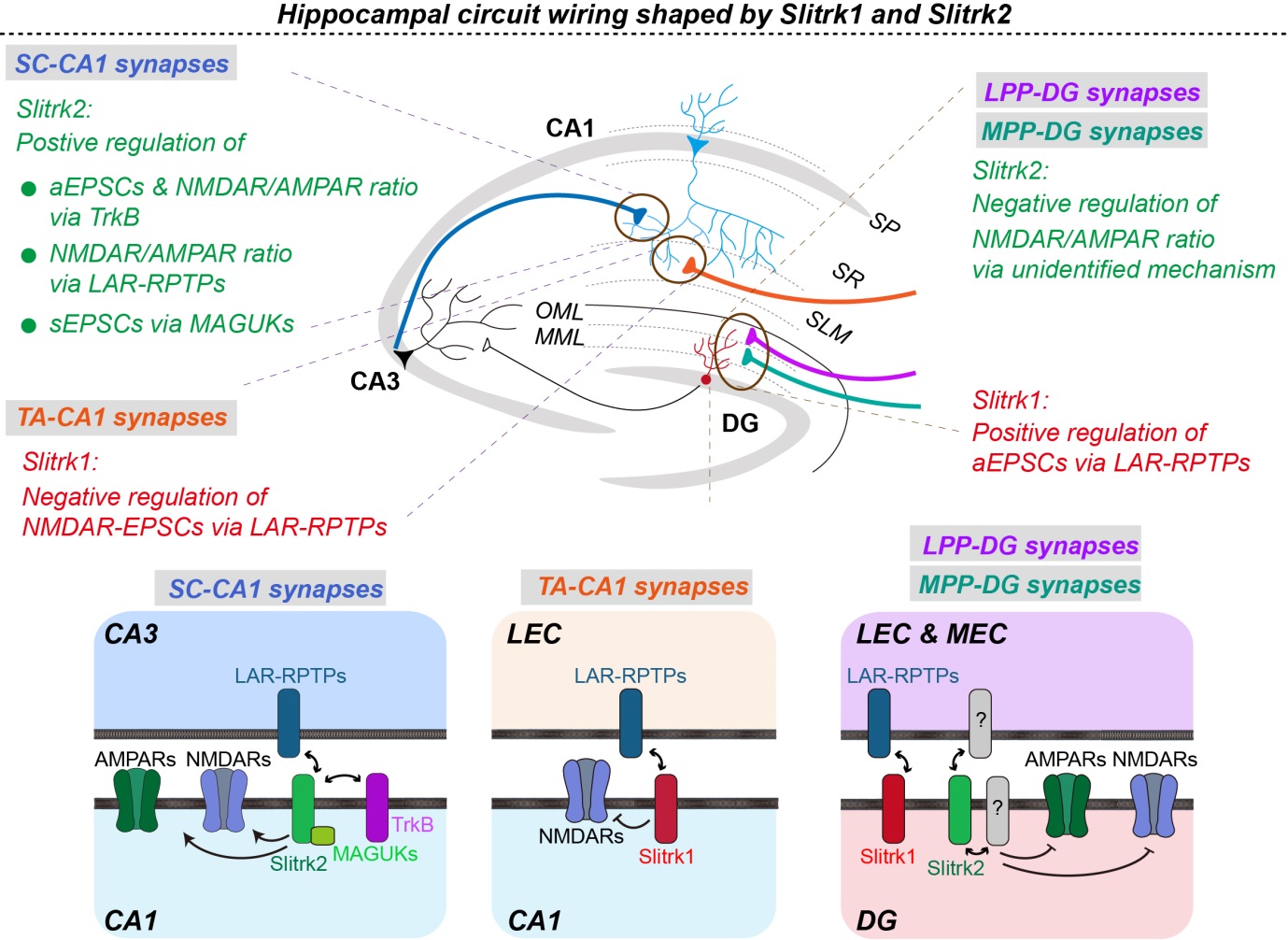
S****17 Fig. Model for actions of postsynaptic Slitrk1 and Slitrk2 in mediating specification of excitatory synaptic properties in the hippocampal circuits via distinct extracellular and intracellular mechanisms.**


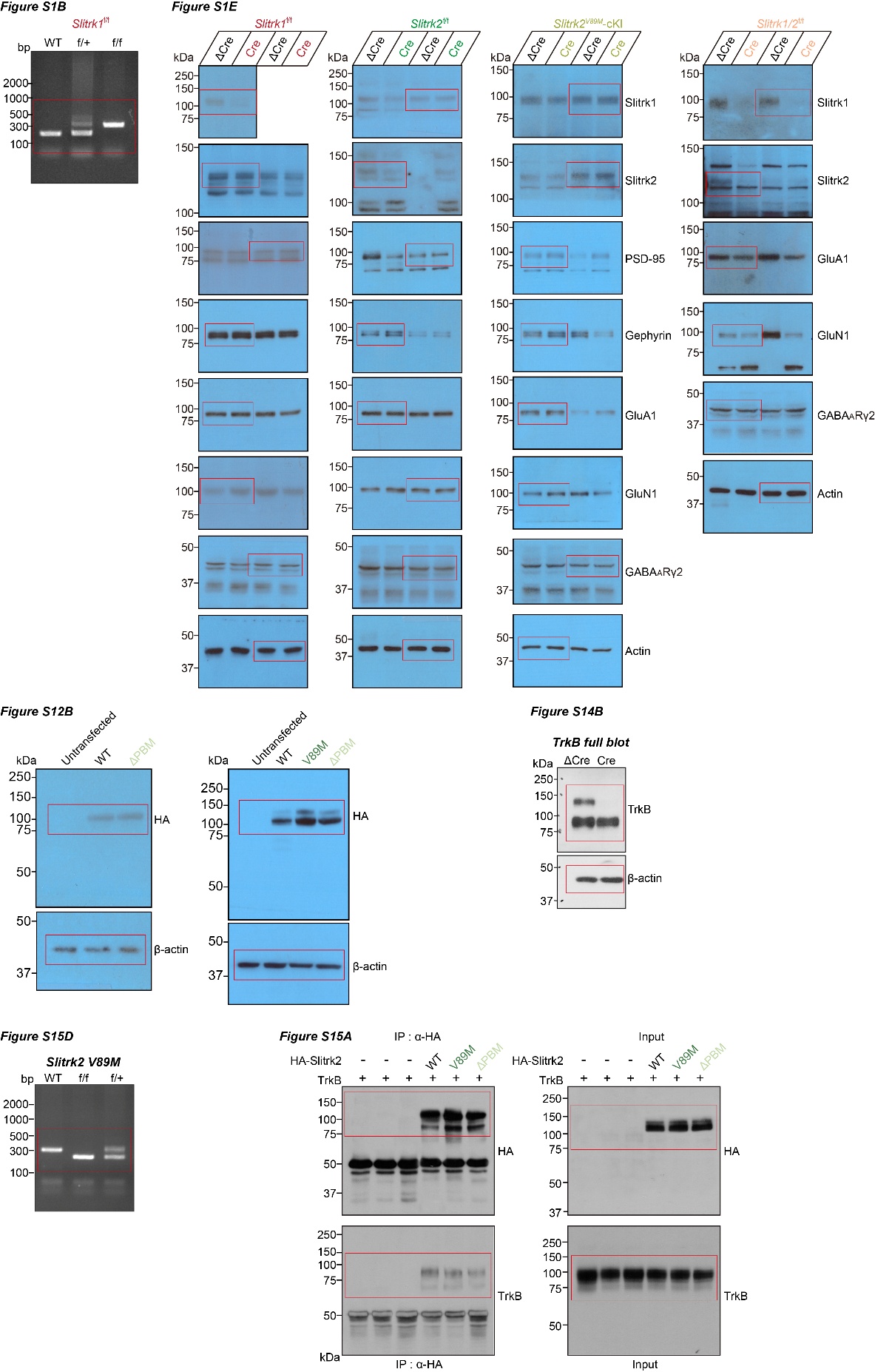


**S18 Fig. Uncropped scanned images of representative immunoblots and agarose gels in the current study.**

**S1 Table. Summary of electrophysiological phenotypes from other knockout models that depict specification of excitatory synaptic properties**

| **Parameters**  **(target circuit)** | *Lphn3*-cKO  [PMID:30792275] | *Ntgn1*-KO  [PMID:25411505] | *Ntgn2*-KO  [PMID:25411505] | *Slitrk1*-cKO  [This paper] | *Slitrk2*-cKO  [This paper] |
| --- | --- | --- | --- | --- | --- |
| sEPSCs  (CA1) | n.a | n.a | n.a | **Frequency↑** | **Frequency↓** |
| mEPSCs  (CA1) | **Frequency↓** | n.a | n.a | **Frequency↓** | **Frequency↓** |
| eEPSCs  (SC-CA1) | **Amplitude↓** | n.s. | n.s. | n.s. | **Amplitude↓** |
| PPRs  (SC-CA1) | n.s. | n.s. | n.s. | n.s. | n.s. |
| eEPSCs  (TA-CA1) | n.s. | n.s. | n.s. | **NMDAR↑** | n.s. |
| PPRs  (TA-CA1) | n.a. | n.s. | n.s. | n.s. | n.s. |
| mEPSCs  (DG) | n.a. | n.s. | n.s. | n.s. | n.s. |
| eEPSCs  (MPP-DG) | n.a. | n.s. | **Slope↓** | n.s. | **Amplitude↑** |
| PPRs  (MPP-DG) | n.a. | n.s. | **PPRs↑** | n.s. | n.s. |
| eEPSCs  (LPP-DG) | n.a. | n.s. | n.s. | n.s. | **Amplitude↑** |
| PPRs  (LPP-DG) | n.a. | n.s. | n.s. | n.s. | n.s. |

Abbreviations: n.a., not analyzed; n.s., not significant. Note that the study documenting the phenotypes from *Ntgn1*-KO and *Ntgn2*-KO mice performed field EPSP recordings.

**S2 Table. Comparison of electrophysiological phenotypes from two different Slitrk1 loss-of-function approaches**

| **Parameters**  **(target circuits)** | *Slitrk1*-KD (rat)  [PMID:29983322] | *Slitrk1*-cKO (mouse)  [This paper] |
| --- | --- | --- |
| sEPSCs | **Frequency↑** | **Frequency↑** |
| AMPAR-eEPSCs  (SC-CA1) | n.s. | n.s. |
| NMDAR-eEPSCs  (SC-CA1) | n.s. | n.s. |
| PPRs  (SC-CA1) | n.s. | n.s. |
| Cumulative charges  (SC-CA1) | **↑** | n.a. |
| aEPSCs  (SC-CA1) | **Frequency↑** | n.s. |
| AMPAR-eEPSCs  (TA-CA1) | **Amplitude↓** | n.s. |
| NMDAR-eEPSCs  (TA-CA1) | n.s. | **Amplitude↑** |
| PPRs  (TA-CA1) | n.s. | n.s. |
| Cumulative charges  (TA-CA1) | **↓** | n.a |
| aEPSCs  (TA-CA1) | **Amplitude↓** | n.s. |

Abbreviations: n.a., not analyzed; n.s., not significant

**S3 Table. Summary of ultrastructural phenotypes from the current study**

| **Parameters**  **(target circuit)** | *Slitrk1*-KD (rat)  [PMID:29983322] | *Slitrk1*-cKO  [This paper] | *Slitrk2*-cKO  [This paper] |
| --- | --- | --- | --- |
| Synaptic structure  [AZ, PSD, SC]  (SC-CA1) | AZ length n.s.  PSD length n.s.  Synaptic cleft width n.a. | **PSD length↑** | n.s. |
| Total vesicle density  (SC-CA1) | **↑** | n.s. | **↓** |
| Docked vesicle density  (SC-CA1) | **↑** | **↓** | **↓** |
| Synaptic structure  [AZ, PSD, synaptic cleft]  (TA-CA1) | n.a. | n.s. | n.s. |
| Total vesicle density  (TA-CA1) | n.a. | n.s. | n.s. |
| Docked vesicle density  (TA-CA1) | n.a. | n.s. | n.s. |
| Synaptic structure  [AZ, PSD, synaptic cleft]  (MPP-DG) | n.a. | n.s. | n.s. |
| Total vesicle density  (MPP-DG) | n.a. | n.s. | n.s. |
| Docked vesicle density  (MPP-DG) | n.a. | n.s. | n.s. |
| Synaptic structure  [AZ, PSD, synaptic cleft]  (LPP-DG) | n.a. | n.s. | n.s. |
| Total vesicle density  (LPP-DG) | n.a. | **↓** | n.s. |
| Docked vesicle density  (LPP-DG) | n.a. | n.s. | n.s. |

Abbreviations: n.a., not analyzed; n.s., non-significant **S4 Table. Summary of electrophysiological phenotypes from the current study**

| **Parameters**  **(target circuit)** | *Slitrk1*-cKO | | *Slitrk2*-cKO | | | *Slitrk1/2*-dcKO | *Slitrk2*^V89M^-cKI | *Ntrk2*-cKO |
| --- | --- | --- | --- | --- | --- | --- | --- | --- |
|  | cKO | +PBM | cKO | +PBM | +ΔPDZ |  |  |  |
| Excitability  (CA1) | **↑** | n.a | **↓** | n.a. | n.a. | n.s. | **↓** | n.s. |
| sEPSCs  (CA1) | **Freq↑** | **Freq↑** | **Freq↓** | **Rescued** | **Freq↓** | n.s. | **Freq↓** | n.s. |
| mEPSCs  (CA1) | **Freq↓** | n.a. | **Freq↓** | n.a. | n.a. | n.a. | n.a. | n.a. |
| eEPSCs  (SC-CA1) | n.s. | n.a. | **AMPAR↓**  **NMDAR↓** | **AMPAR↓**  **NMDAR↓** | **Rescued** | **AMPAR↓**  **NMDAR↓** | n.s. | **AMPAR↓**  **NMDAR↓** |
| PPRs  (SC-CA1) | n.s. | n.a. | n.s. | n.a. | n.a. | n.s. | n.a. | n.a. |
| aEPSCs  (SC-CA1) | n.s. | n.a. | **Freq↓** | **Rescued** | **Rescued** | **Freq↓** | **Freq↓** | **Freq↓** |
| eEPSCs  (TA-CA1) | **NMDAR↑** | **NMDAR↑** | n.s. | n.a. | n.a. | **NMDAR↑** | n.a. | n.a. |
| PPRs  (TA-CA1) | n.s. | n.a. | n.s. | n.a. | n.a. | n.s. | n.a. | n.a. |
| aEPSCs  (TA-CA1) | n.s. | n.a. | n.s. | n.a. | n.a. | n.s. | n.a. | n.a. |
| Excitability  (DG) | n.s. | n.a. | n.s. | n.a. | n.a. | n.s. | n.a. | n.a. |
| sEPSCs  (DG) | n.s. | n.a. | n.s. | n.a. | n.a. | n.s. | n.a. | n.a. |
| eEPSCs  (MPP-DG) | n.s. | n.a. | **AMPAR↑**  **NMDAR↑** | **Rescued** | **Rescued** | **AMPAR↑**  **NMDAR↑** | n.s. | n.s. |
| PPRs  (MPP-DG) | n.s. | n.a. | n.s. | n.a. | n.a. | n.a. | n.a. | n.a. |
| aEPSCs  (MPP-DG) | **Freq↓** | **Freq↓** | n.s. | n.a. | n.a. | **Freq↓** | n.a. | n.a. |
| eEPSCs  (LPP-DG) | n.s. | n.a. | **AMPAR↑**  **NMDAR↑** | **Rescued** | **Rescued** | **AMPAR↑**  **NMDAR↑** | n.s. | n.s. |
| PPRs  (LPP-DG) | n.s. | n.a. | n.s. | n.a. | n.a. | n.a. | n.a. | n.a. |
| aEPSCs  (LPP-DG) | **Freq↓** | **Freq↓** | n.s. | n.a. | n.a. | **Freq↓** | n.a. | n.a. |

Abbreviations: n.a., not analyzed; n.s., non-significant
