# Supplementary material for: Paralogs of Slitrk cell adhesion molecules configure excitatory synapse specificity via distinct cellular mechanisms
Source: PLoS Biol. 2025 Dec 18;23(12):e3003576. doi: 10.1371/journal.pbio.3003576 (PMC12742733; doi:10.1371/journal.pbio.3003576)
Supplement: Attachment — Submitted filename: Rebuttal_revision [FINAL].pdf [file pbio.3003576.s028.pdf]

Authors' rebuttal letter for Kim, Kim and Kim *et al.*, "Paralogs of Slitrk cell adhesion molecules configure excitatory synapse specificity via distinct cellular mechanisms", and description of changes made to the revised manuscript

We are grateful to the Editor and Reviewer #1 for their careful evaluation and critical analysis of our manuscript. Our responses to the specific comments of the reviewers are provided below, with the complete reviewers' comments shown in *italic* typeface, and our response in bold typeface.

Note from the Editorial Office

***Title:*
-- We would like to tweak your title to provide a bit more information on the nature of the Slitrk proteins. Is this alternative version acceptable to you? "Paralogs of Slitrk cell adhesion molecules configure excitatory synapse specificity via distinct cellular mechanisms"
We accept your suggestion and have updated the title in the revised manuscript accordingly.

***Financial disclosure statement:*
-- Please include whether the sponsors or funders played a role in the study design, data collection and analysis, decision to publish, or preparation of the manuscript. Please also include links to the funding agencies.
We have updated the financial disclosure statement in the revised manuscript to clearly state the funder's roles and have included the required links to the funding agencies.

***Ethics:*
-- The Ethics statement needs to be a separate, independent (and the first) subheading in the Material & Methods section. Please also include the national or international origin of the guide of care that you adhered to.
We have moved the Ethics statement to the very first subheading of the Materials and Methods section and have specified the relevant national/international guidelines of care we adhered to.

***Data:*
-- Thank you for including the numerical data supporting the figures. I looked at some of the values shown in Data S1 and noticed a possible inconsistency related to Fig S16. It seems unclear whether the presented values are all normalized to the mean of the deltaCre value or not. In the numerical data, the mean for the Slitrk1 deltaCre is 0.905, but this looks different to what is shown in the figure (the value appears to be close to 1.0). In contrast, the Slitrk2-5 deltaCre are all extremely close to 1.0, suggesting they were normalized, and this does match what is shown in the figure. Please double-check this, and also confirm that the other numerical data are correct.
We thank you for carefully checking the data. We confirmed that the data plotted in S16 Fig

were indeed correctly normalized to the ΔCre (approaching 1.0). However, we found that the corresponding values listed in the Data S1 file were inadvertently the pre-normalization values. We have now corrected Data S1 to list the normalized values, ensuring consistency with the figure. We have also re-verified that all other numerical datasets in Data S1 are correct and consistent with the figures.

***Gels:*
-- Thank you for providing uncropped images of gels in S18 Fig. We noticed that there are other gel images in additional figures, including S12BG, S14B, and S15AD. Please confirm that you have included the original, uncropped, and minimally adjusted images for all of the blots and gels. Our guidelines for how to prepare and upload this data are available here: https://journals.plos.org/plosbiology/s/figures#loc-blot-and-gel-reporting-requirements
We have ensured that all relevant raw images are provided. Regarding the specific figures mentioned (S12BG, S14B, and S15AD), we realized that the uncropped images were already included in the previous version of S18 Fig, but they were mislabeled with incorrect figure numbers. We have now corrected the labeling in S18 Fig to clearly indicate the corresponding panels. We also confirmed that the raw data for S12G is included in S18 Fig.

To submit your revision, please go to https://www.editorialmanager.com/pbiology/ and log in as an Author. Click the link labelled 'Submissions Needing Revision' to find your submission record. Your revised submission must include the following:
- a cover letter that should detail your responses to any editorial requests, if applicable, and whether changes have been made to the reference list
- a Response to Reviewers file that provides a detailed response to the reviewers' comments (if applicable, if not applicable please do not delete your existing 'Response to Reviewers' file.)
- a track-changes file indicating any changes that you have made to the manuscript.
We have followed these instructions. We have uploaded the revised manuscript (both clean and track-changed versions), a detailed Response to Reviewers file, and a coverletter addressing the editorial requests.

NOTE: If Supporting Information files are included with your article, note that these are not copyedited and will be published as they are submitted. Please ensure that these files are legible and of high quality (at least 300 dpi) in an easily accessible file format. For this reason, please be aware that any references listed in an SI file will not be indexed. For more information, see our Supporting Information guidelines: https://journals.plos.org/plosbiology/s/supporting-information
We have reviewed the Supporting Information files to ensure that they meet the quality and formatting requirements.

1. A 'Response to Reviewers' file - this should detail your responses to the editorial requests, present a point-by-point response to all of the reviewers' comments, and indicate the changes made to the manuscript.
**NOTE: In your point-by-point response to the reviewers, please provide the full context of each review. Do not selectively quote paragraphs or sentences to reply to. The entire set of reviewer comments should*

be present in full and each specific point should be responded to individually, point by point. You should also cite any additional relevant literature that has been published since the original submission and mention any additional citations in your response.
We have prepared this point-by-point response letter to address all comments in full.

Published Peer Review History
Please note that you may have the opportunity to make the peer review history publicly available. The record will include editor decision letters (with reviews) and your responses to reviewer comments. If eligible, we will contact you to opt in or out. Please see here for more details: https://plos.org/published-peer-review-history/
We have noted the policy regarding the Published Peer Review History.

Press
Should you, your institution's press office or the journal office choose to press release your paper, please ensure you have opted out of Early Article Posting on the submission form. We ask that you notify us as soon as possible if you or your institution is planning to press release the article.
We have noted the press release policy.

Protocols deposition
To enhance the reproducibility of your results, we recommend that if applicable you deposit your laboratory protocols in protocols.io, where a protocol can be assigned its own identifier (DOI) such that it can be cited independently in the future. Additionally, PLOS ONE offers an option for publishing peer-reviewed Lab Protocol articles, which describe protocols hosted on protocols.io. Read more information on sharing protocols at https://plos.org/protocols?utm_medium=editorial-email&utm_source=authorletters&utm_campaign=protocols
We appreciate the recommendation and have reviewed the options for protocol deposition.

Reviewers' comments:

Reviewer #1: The authors have addressed the remaining points to further improve their study, and are transparent about all results and project designs.
We are grateful to Reviewer 1 for his/her positive evaluation and endorsement of our manuscript.

We again thank the Editor and reviewers for their careful assessment of our paper and hope that the revised manuscript is now acceptable for publication in *PLOS Biology*.
