# Supplementary material for: Paralogs of Slitrk cell adhesion molecules configure excitatory synapse specificity via distinct cellular mechanisms
Source: PLoS Biol. 2025 Dec 18;23(12):e3003576. doi: 10.1371/journal.pbio.3003576 (PMC12742733; doi:10.1371/journal.pbio.3003576)
Supplement: Attachment — Submitted filename: Rebuttal [FINAL].doc [file pbio.3003576.s026.doc]

**Authors’ rebuttal letter for Kim, Kim and Kim *et al*., “Excitatory Slitrk paralogs configure synaptic specificity via distinct extracellular and intracellular mechanisms”, and description of changes made to the revised manuscript**

**We are grateful to the reviewers for their careful evaluation and critical analysis of our manuscript. Their extensive comments have identified weaknesses in our previous arguments and inadequate explanations of experiments and highlighted where more data were needed. To fully respond to the reviewers’ comments as carefully as possible, we performed an extensive series of additional experiments and modified the presentation of the manuscript. Our responses to the specific comments of the reviewers are provided below, with the complete reviewers’ comments shown in *italic* typeface, our response in bold typeface, and the cited text in bold blue typeface.**

**Note from the Editorial Office**

*1. A 'Response to Reviewers' file - this should detail your responses to the editorial requests, present a point-by-point response to all of the reviewers' comments, and indicate the changes made to the manuscript.*

**NOTE: In your point-by-point response to the reviewers, please provide the full context of each review. Do not selectively quote paragraphs or sentences to reply to. The entire set of reviewer comments should be present in full and each specific point should be responded to individually, point by point. You should also cite any additional relevant literature that has been published since the original submission and mention any additional citations in your response.*

**We followed the instructions provided by the Editorial Office by tailoring our rebuttal letter to provide detailed responses to the full text of each comment and indicate relevant changes in the revised manuscript.**

*2. In addition to a clean copy of the manuscript, please also upload a 'track-changes' version of your manuscript that specifies the edits made. This should be uploaded as a "Revised Article with Changes Highlighted" file type.*

**As instructed, we uploaded both changes-tracked and clean copies of the manuscript.**

**Re-submission Checklist**

*When you are ready to resubmit your revised manuscript, please refer to this re-submission checklist:* [*https://plos.io/Biology_Checklist*](https://plos.io/Biology_Checklist)

*To submit a revised version of your manuscript, please go to* [*https://www.editorialmanager.com/pbiology/*](https://www.editorialmanager.com/pbiology/) *and log in as an Author. Click the link labelled 'Submissions Needing Revision' where you will find your submission record.*

**We referred to this checklist and ensured that all materials were uploaded.**

*Please make sure to read the following important policies and guidelines while preparing your revision:*

**Published Peer Review**

*Please note while forming your response, if your article is accepted, you may have the opportunity to make the peer review history publicly available. The record will include editor decision letters (with reviews) and your responses to reviewer comments. If eligible, we will contact you to opt in or out. Please see here for more details:* [*https://blogs.plos.org/plos/2019/05/plos-journals-now-open-for-published-peer-review/*](https://blogs.plos.org/plos/2019/05/plos-journals-now-open-for-published-peer-review/)

**PLOS Data Policy**

*Please note that as a condition of publication PLOS' data policy (*[*http://journals.plos.org/plosbiology/s/data-availability*](http://journals.plos.org/plosbiology/s/data-availability)*) requires that you make available all data used to draw the conclusions arrived at in your manuscript. If you have not already done so, you must include any data used in your manuscript either in appropriate repositories, within the body of the manuscript, or as supporting information (N.B. this includes any numerical values that were used to generate graphs, histograms etc.). For an example see here: [http://www.plosbiology.org/article/info%3Adoi%2F10.1371%2Fjournal.pbio.1001908#s5](http://www.plosbiology.org/article/info%3Adoi%2F10.1371%2Fjournal.pbio.1001908#s5)
*Blot and Gel Data Policy**

*We require the original, uncropped and minimally adjusted images supporting all blot and gel results reported in an article's figures or Supporting Information files. We will require these files before a manuscript can be accepted so please prepare them now, if you have not already uploaded them. Please carefully read our guidelines for how to prepare and upload this data: [https://journals.plos.org/plosbiology/s/figures#loc-blot-and-gel-reporting-requirements](https://journals.plos.org/plosbiology/s/figures#loc-blot-and-gel-reporting-requirements)
*Protocols deposition**

*To enhance the reproducibility of your results, we recommend that if applicable you deposit your laboratory protocols in* [*protocols.io*](http://protocols.io/)*, where a protocol can be assigned its own identifier (DOI) such that it can be cited independently in the future. Additionally, PLOS ONE offers an option for publishing peer-reviewed Lab Protocol articles, which describe protocols hosted on* [*protocols.io*](http://protocols.io/)*. Read more information on sharing protocols at* [*https://plos.org/protocols?utm_medium=editorial-email&utm_source=authorletters&utm_campaign=protocols*](https://plos.org/protocols?utm_medium=editorial-email&utm_source=authorletters&utm_campaign=protocols)

**We reviewed the various policies of PloS Biology and followed them in the revised manuscript. To comply with the ‘Blot and Gel Data Policy’, we now present Supplementary Fig. 18 in the revised manuscript, which includes the original/uncropped versions of ALL images presented in the current study.**

**Reviewers' comments:**

*Reviewer #1: Functional differences of paralogs of synaptic cell adhesion molecules and their circuit-dependent roles remain incompletely understood. Focusing on the LRR superfamily members Slitrk1 and Slitrk2, Kim and colleagues use cKO mice for their analyses. They apply immunohistochemistry to demonstrate that Slitrk1 and Slitrk2 have distinct expression patterns across the adult mouse hippocampus. Slice electrophysiology and TEM demonstrate that loss of Slitrk1 or Slitrk2 in hippocampal neurons affects their intrinsic excitability, synaptic transmission, and synaptic vesicle density in a circuit-dependent manner. The authors then use slice electrophysiology to demonstrate that extra- and intra-cellular Slitrk1 and Slitrk2 interaction domains differently affect synaptic transmission. This study is a tour de force and presents extensive electrophysiology and TEM data for Slitrk1 and Slitrk2 and their mutants. The results provide evidence that these paralogs can play synapse- and circuit-specific roles. The authors also demonstrate the importance of specific binding domains of Slitrk1 and Slitrk2 for synaptic transmission. However, several results require additional controls, and in several cases, the data do not fully support some experimental conclusions and statements. This raises a comparatively large number of points, as provided below.*

**We appreciate the reviewer’s appraisal of our study, their careful analysis of the original manuscript, and the constructive comments that helped us improve our manuscript. We hope that our additional efforts and revisions completely address the reviewer’s concerns.**

*Major points*

*1. Potential changes in synapse number in the cKO mice and Slitrk mutants need to be addressed. This is required to assess their synaptic function and interpret electrophysiological analyses. This important analysis should, at a minimum, be performed in the cKO mice and could e.g. be addressed by filling CA1 and DG neurons with dye and quantifying dendritic spine number.*

**We agree that, for the transgenic mouse lines generated for the current study, the reader needs data on the synapse numbers to properly interpret the extensive electrophysiological recordings described in the original manuscript. To address this, we performed immunohistochemical analyses in hippocampal CA1 and DG layers from adult *Slitrk1*-cKO or *Slitrk2*-cKO mice. We found that postsynaptic deletion of Slitrk1 or Slitrk2 similarly reduced the number of excitatory synaptic puncta in the CA1 but not in the DG. These results suggest that both Slitrk1 and Slitrk2 contribute to maintaining excitatory synapse density, in line with the reduced mEPSC frequency seen in the CA1 pyramidal neurons of the respective *Slitrk*-cKO mice. In contrast, Slitrk2, but not Slitrk1, is not required for maintenance of excitatory synapse density in the DG granule neurons. These new results are presented in Supplementary Figures 8 and 9 of the revised manuscript.**

*2. The authors show that CA1 neurons in the Slitrk1 cKO, the Slitrk2 cKO, and the Slitrk2 V98M mutant have altered intrinsic excitability (Supp. Fig. 2). This suggests that these mutations have synapse-independent effects on the electrophysiological properties of these cells. This effect on excitability may impact synaptic transmission. The authors need to address this concern.*

**These are excellent points, and we acknowledge that we did not examine whether the altered intrinsic excitabilities of CA1 pyramidal neurons lacking each Slitrk paralog or knocked in with Slitrk2 V89M could contribute to the altered excitatory synaptic transmission. To address this possibility, we employed DREADD–based chemogenetics to either boost (i.e., hM3Dq) or dampen (i.e., hM4Di) the excitability of CA1 pyramidal neurons from the indicated *Slitrk1*-cKO or *Slitrk2*-V89M mice. We first confirmed that the DREADD strategy successfully normalized the altered excitability seen in the Slitrk transgenic mice, as assessed by electrophysiological analyses (now presented in Supplementary Figure 5 of the revised manuscript). We then measured sEPSCs in the CA1 pyramidal neurons from each Slitrk transgenic mouse. We found that normalizing the altered excitability does NOT influence the phenotypes presented in the original manuscript (i.e., increased sEPSC frequency for *Slitrk1*-cKO mice, decreased sEPSC frequencies for *Slitrk2*-cKO and *Slitrk2*-V89M mice). These results suggest that the alterations in spontaneous excitatory synaptic transmission induced by conditional deletion of postsynaptic Slitrk paralogs are not derived from altered intrinsic excitabilities. These new results are presented in Supplementary Figure 6 of the revised**
**manuscript.**

*3. The authors state that the pathogenic Slitrk2 V89M mutant affects CA1 micro-circuits but not the DG. However, this is based on the finding that the Slitrk2 V98M mutant affects CA1 spontaneous EPSCs and SC-CA1 asynchronous EPSCs, while these parameters are not measured in DG neurons or synapses. Without analyzing the same parameters in both CA1 and DG micro-circuits, the data does not sufficiently support that the mutant plays a micro-circuit specific role.*

**We did not analyze DG microcircuits because we failed to detect any alteration of excitatory synaptic properties in the DG granule neurons of *Slitrk2*-cKO mice (as presented in Figure 3 of the original manuscript). We hope that this fully explains why we did not include electrophysiological analyses performed on microcircuits in DG granule neurons of *Slitrk2*-cKO mice.**

*Minor points*

*4. Related to point 3, there are several other instances in the manuscript when only some of the electrophysiology analyses are shown for specific Slitrk mutants (e.g., for the PDZ mutants in Fig. 6, synchronous and asynchronous EPSCs are not analyzed for TA-CA1 synapses, and spontaneous EPSCs are not analyzed for DG granule neurons). This makes it challenging to assess cell- and circuit-specific effects, which should, at a minimum, be discussed.*

**We apologize that we did not sufficiently explain these experiments in the original manuscript. In the studies presented in Figures 2 and 3 of the original manuscript, we documented various electrophysiological phenotypes in *Slitrk*-cKO mice devoid of Slitrk expression in postsynaptic areas of various microcircuits (i.e., CA1: SC-CA1 vs. TA-CA1; DG: MPP-DG vs. LPP-DG). Thus, for the rescue experiments documented in Figure 5 and 6, we focused on synapses, in which we had observed that each *Slitrk*-cKO triggered prominent alterations in the excitatory synaptic properties.**

*5. The control baseline values for electrophysiology recordings differ in part significantly between experiments (e.g. Fig.2D vs 2F frequency, Fig. 2N vs 2P frequency, Fig. 3H vs 3J EPSC I-O slope, Fig. 5D vs 2F frequency). Can the authors explain this discrepancy?*

**We appreciate the reviewer’s careful observation. Ideally, the baseline frequencies obtained in the control groups should be perfectly comparable. However, previous relevant studies reported that slightly different values (1.2~3.2-fold differences) often occur across different experiments, even within the same study (see our summary table below, which is included for the reviewer’s reference). In addition, it is possible that injections of AAVs expressing ΔCre into different transgenic mouse lines could yield slightly different control values in different recording experiments. We hope that these explanations assuage the reviewer’s concerns.**

***Table R1. Summary of electrophysiological parameters in control groups of previous reports***

| **Paper** | | | **Parameter/region** | **Value** | | | **Variation**  **(fold-changes)** |
| --- | --- | --- | --- | --- | --- | --- | --- |
| Ref #1 | PMID | 36344520 | sEPSC frequency/dentate gyrus | **Figure 4-e)** | **Figure 5-d )** | **Figure 5-f )** | 2.0 ~ 2.5 |
|  |  |  |  | Ctrl ) 1.0 Hz | Pre ) 2.5 Hz | Off ) 2.0 Hz |  |
| Ref #2 | PMID | 28521135 | MNTB neuron  sEPSC/Amp. | **Figure 1-a )** | **Figure 2-f )** |  | 1.25 |
|  |  |  |  | Ctrl ) 28 pA | Ctrl ) 35 pA |  |  |
| Ref #3 | PMID | 33646123 | CA1(SC pathway)  eEPSC/IO slope | **Figure 4-c )** | **Figure 5-c )** |  | 1.2 |
|  |  |  |  | Ctrl ) 12 | Ctrl ) 10 |  |  |
| Ref #4 | PMID | 33646123 | CA1(PP pathway)  eEPSC/IO slope | **Figure 4-f )** | **Figure 5-f )** |  | 1.3 |
|  |  |  |  | Ctrl ) 5 | Ctrl ) 3.8 |  |  |
| Ref #5 | PMID | 37591863 | Cerebellum  mIPSC/Amp. | **Figure 6-a,c )** | **Figure 6-b )** |  | 1.3 |
|  |  |  |  | Ctrl ) 100 pA | Ctrl ) 75 pA |  |  |
| Ref #6 | PMID | 39506118 | DG / Astrocytes  sEPSC/Freq. | **Figure 3-j )** | **Ext Fig 4-d )** |  | 3.2 |
|  |  |  |  | Ctrl ) 0.25 Hz | Ctrl ) 0.8 Hz |  |  |

*6. Interpreting the high-resolution immunostaining data requires specificity controls for the antibodies used. The authors can use knockout controls for Slitrk1/2 immunostaining with eMAP expanded tissue to ensure antibody specificity is maintained with this technique.*

**We agree with the reviewer’s comment and performed additional experiments with eMAP-expanded *Slitrk1*-cKO and *Slitrk2*-cKO tissues. We found that each Slitrk deletion reduced the number of Slitrk immunofluorescence signals that were colocalized with postsynaptic markers (SHANK2 and PSD-95). Note that the eMAP experiments specifically captured immunofluorescent signals from synaptic sites (not soma), including those of neurons that projected onto virus-injected areas, so the suppression of each Slitrk antibody signal was not marked as those seen in unexpanded tissues. We have now included these new results in Figure 1 and Supplementary Figure 2 of the revised manuscript and revised the Results and Methods sections accordingly.**

*7. The authors can be commended for the controls of their mutant mice (Fig. S1, Fig. S6). But to what extent are the PBM, V89M, and PDZ mutants expressed at synapses? They could consider expressing the constructs in cultured neurons and assess whether the mutant proteins are properly sorted to synapses.*

**In response to the reviewer’ suggestion, we performed immunocytochemical analyses in cultured hippocampal neurons transfected with the Slitrk2 expression vectors (Slitrk2 WT, Slitrk2 PBM, Slitrk2 ΔPDZ, or Slitrk2 V89M). We found that all of the examined Slitrk2 variants were targeted to excitatory synapses (labeled by SHANK^+^ immunoreactive puncta in MAP2-labeled dendrites). These results, which were comparable to those obtained with Slitrk2 WT, suggest that the binding activities of Slitrk2 to LAR-RPTPs or PDZ proteins are not involved in the excitatory synapse targeting of Slitrk2 variants in cultured hippocampal neurons. These new results are presented in Supplementary Figure 13 of the revised manuscript.**

*8. Can the authors describe how they could precisely and separately stimulate axon tracts that run very close to each other (e.g., MPP and LPP)?*

**The information requested by the reviewer was described in detail in the Methods section of the original manuscript as follows: “***For selective activation of MPP or LPP afferents in the DG, monopolar stimulation electrodes were positioned based on the established laminar organization of these specific pathways. We verified input identity by short-term plasticity signatures: MPP stimulation induced paired-pulse depression, whereas LPP stimulation elicited paired-pulse facilitation, as previously described (Petersen et al., 2013)***”*.* Our laboratory has used this electrical stimulation strategy in many projects, some of which were previously published (e.g., PMID: 35022233).**

*9. The Barnes Maze test was only performed with mice in which Slitrk2 V89M is expressed by CA1 neurons. Comparative data across lines may have helped to interpret circuit-specific roles better. This limitation should be mentioned.*

**Our intention using Barnes Maze test was to allow us to compare our present model with our previous results (PMID: 35840571) demonstrating that the conditional deletion of Slitrk2 in hippocampal CA1 neurons impairs spatial reference memory in adult mice. Indeed, our present results showed that conditional KI of Slitrk2 V89M in the hippocampal CA1 recapitulates the Slitrk2 loss-of-function behavioral phenotype. As we did not detect any other abnormality in behavioral assays performed using CA1-specific *Slitrk2*-cKO mice, besides that observed in the Barnes Maze test, we did not pursue any other behavioral paradigm in the current study. We hope that the reviewer understands our rationale for performing the Barnes Maze test using CA1-specific *Slitrk2*-V89M KI and concurs with us.**

*10. For statistical analyses, are replicates considered to be each cell/synapse or each mouse? This is unclear from the methods, and there are a few places (e.g., Fig. 1J-M figure legend) where the sampling is unclear.*

**To address the reviewer’s concern, we now note the numbers of biological replicates in ALL figure legends of the revised manuscript.**

*11. The summary tables in the supplement are very helpful. They could also include the PBM and PDZ mutant data.*

**To accommodate this excellent suggestion, we included information on Slitrk2 PBM and Slitrk2 ΔPDZ in Supplementary Table 4 of the revised manuscript.**

| **Parameters**  **(target circuit)** | *Slitrk1*-cKO | | *Slitrk2*-cKO | | | *Slitrk1/2*-dcKO | *Slitrk2*^V89M^-cKI | *Ntrk2*-cKO |
| --- | --- | --- | --- | --- | --- | --- | --- | --- |
|  | cKO | +PBM | cKO | +PBM | +ΔPDZ |  |  |  |
| Excitability  (CA1) | **↑** | - | **↓** | - | - | n.s. | **↓** | n.s. |
| sEPSCs  (CA1) | **Freq↑** | **Freq↑** | **Freq↓** | **Rescued** | **Freq↓** | n.s. | **Freq↓** | n.s. |
| mEPSCs  (CA1) | **Freq↓** | n.a | **Freq↓** | n.a | n.a | n.a | n.a | n.a |
| eEPSCs  (SC-CA1) | n.s. | - | **AMPAR↓**  **NMDAR↓** | **AMPAR↓**  **NMDAR↓** | **Rescued** | **AMPAR↓**  **NMDAR↓** | n.s. | **AMPAR↓**  **NMDAR↓** |
| PPRs  (SC-CA1) | n.s. | - | n.s. | - | - | n.s. | n.a. | n.a. |
| aEPSCs  (SC-CA1) | n.s. | - | **Freq↓** | **Rescued** | **Rescued** | **Freq↓** | **Freq↓** | **Freq↓** |
| eEPSCs  (TA-CA1) | **NMDAR↑** | **NMDAR↑** | n.s. | - | - | **NMDAR↑** | n.a. | n.a. |
| PPRs  (TA-CA1) | n.s. | - | n.s. | - | - | n.s. | n.a. | n.a. |
| aEPSCs  (TA-CA1) | n.s. | - | n.s. | - | - | n.s. | n.a. | n.a. |
| Excitability  (DG) | n.s. | - | n.s. | - | - | n.s. | n.a. | n.a. |
| sEPSCs  (DG) | n.s. | - | n.s. | - | - | n.s. | n.a. | n.a. |
| eEPSCs  (MPP-DG) | n.s. | - | **AMPAR↑**  **NMDAR↑** | **Rescued** | **Rescued** | **AMPAR↑**  **NMDAR↑** | n.s. | n.s. |
| PPRs  (MPP-DG) | n.s. | - | n.s. | - | - | n.a. | n.a. | n.a. |
| aEPSCs  (MPP-DG) | **Freq↓** | **Freq↓** | n.s. | - | - | **Freq↓** | n.a. | n.a. |
| eEPSCs  (LPP-DG) | n.s. | - | **AMPAR↑**  **NMDAR↑** | **Rescued** | **Rescued** | **AMPAR↑**  **NMDAR↑** | n.s. | n.s. |
| PPRs  (LPP-DG) | n.s. | - | n.s. | - | - | n.a. | n.a. | n.a. |
| aEPSCs  (LPP-DG) | **Freq↓** | **Freq↓** | n.s. | - | - | **Freq↓** | n.a. | n.a. |

*12. The authors should discuss the unexpected presynaptic localization of Slitrk1/2 and how this fits in with their other data.*

**Although we cannot exclude the possibility that a proportion of Slitrk1 and Slitrk2 proteins are localized to presynaptic sites, none of our experiments demonstrated the presynaptic localization of these Slitrk paralogs. We hope that the reviewer accepts this clarification.**

*13. The Slitrk2 LAR-RPTP and PDZ binding domain mutants each have one electrophysiological property changed compared to baseline (LAR-RPTP: SC-CA1 synchronous EPSCs, PDZ: spontaneous EPSCs). However, the authors conclude that "LAR-RPTP engages with Slitrk1, not with Slitrk2, in regulating synaptic specificity" and "Slitrk2 binding to PDZ domain-containing proteins is required for its regulation of specific excitatory synapse properties". It is unclear why the authors have emphasized the importance of one Slitrk2 binding domain over the other when the data suggests that both are important.*

**We thank the reviewer for pinpointing these potentially misleading statements in the original manuscript. As the reviewer’s interpretation is correct, we modified the indicated sentences as follows: “***LAR-RPTPs engage with Slitrk1, but not with Slitrk2, in regulating specification of excitatory synaptic properties (except for eEPSCs at SC-CA1 synapses) in hippocampal microcircuits***”***.* **Moreover, we added the following sentence to the revised manuscript to emphasize our rationale for using various Slitrk2 variants in our molecular replacement experiments: “***Although the interaction of Slitrk2 with LAR-RPTPs is required to control eEPSCs at SC-CA1 synapses, the other excitatory synaptic properties governed by Slitrk2 are not dictated by this interaction***”***.*

*14. The Results section describing the Slitrk1/2 double knockout states that "This suggests that both Slitrk1 and Slitrk2 are functionally non-redundant in regulating a variety of excitatory synaptic properties in hippocampal subfields, but operate via the same pathways in regulating basal synaptic transmission and intrinsic excitability in CA1 pyramidal neurons". The authors argue elsewhere in the paper (Fig. 5, 6) that these paralogs operate through different binding domains and thus different pathways. This discrepancy should be resolved.*

**We thank the reviewer for pinpointing this potentially confusing description of our experimental results in the original manuscript. In the revised manuscript, the indicated sentences have been corrected as follows: “***This suggests that Slitrk1 and Slitrk2 are functionally non-redundant in regulating various excitatory synaptic properties in the hippocampal DG microcircuits, but likely operate via convergent pathways in regulating basal synaptic transmission and intrinsic excitability in CA1 pyramidal neurons, as shown by the opposite phenotypes produced by loss of each Slitrk paralog***”.**

*15. The Discussion states that "(…) the Slitrk2V89M mutation recapitulates the effects of Slitrk2-cKO on spontaneous and asynchronous, but not synchronous, synaptic transmission in hippocampal circuits. These results suggest that Slitrk2V89M acts as either a loss-of-function or a gain-of-function mutation, depending on the hippocampal microcircuit involved." It is unclear how these data suggest a gain-of-function role of the V89M mutation. Instead, these results may be due to the Slitrk2 V89M protein retaining some of its endogenous functional capacity. This should be discussed.*

**We apologize for imprecisely describing the nature of Slitrk2^V89M^ in the indicated sentence of the original manuscript. The phenotypes we documented using *Slitrk2*-cKO mice were partly recapitulated in the corresponding synapse types of *Slitrk2*-V89M cKI mice, indicating that the Slitrk2 V89M mutation does not inhibit the endogenous Slitrk2 protein function in a subset of these synapses. The reviewer is correct that it is reasonable to conclude that Slitrk2 V89M retains the functional capacity of endogenous Slitrk2. Thus, we changed the indicated sentence in the revised manuscript to read: “***These results suggest that Slitrk2V89M acts as a loss-of-function mutation in the hippocampal microcircuit, where postsynaptic loss of Slitrk2 exerts profound deficits in excitatory synaptic properties***”.**

*16. This study assesses the role of Slitrks in mature synapses, not in development. This is important and can be stated in the text. This line should be changed in the Discussion: "Notwithstanding these differences, our study reinforces the idea that [...] an individual protein might perform distinct roles during development."*

**We agree with the reviewer’s comment and have changed the indicated sentence as follows in the revised manuscript: “***Notwithstanding these differences, our study reinforces the idea that cKO analyses are essential for reaffirming the circuit-specific role of a synaptic CAM, and that an individual protein might perform distinct roles at specific developmental stages***”.**

*17. The term "synapse specificity" is referred to in the title and throughout the text of the manuscript. However, this terminology is often used to refer to the specification of synapse formation between distinct neuronal populations. To avoid misunderstanding, the authors can clarify that they refer to the specification of synaptic properties.*

**This is an excellent suggestion. We carefully reviewed the entire manuscript and changed the term “synaptic specificity” or “synapse specificity” to “the specification of synaptic properties” or “the specification of excitatory synaptic properties” in the revised manuscript. We hope the reviewer is satisfied with our action.**

*Reviewer #2: Kim et al. conducted a comprehensive investigation into the functions of Slitrk1 and Slitrk2, two synaptic cell adhesion molecules from the Slitrk family, in regulating excitatory synaptic specificity within hippocampal networks. Utilizing conditional knockout mice alongside electrophysiological recordings, high-resolution imaging, and molecular interaction analyses, the authors sought to determine the distinct, non-redundant roles of these Slitrk paralogs in synaptic function. Their results indicate that Slitrk1 and Slitrk2 influence synaptic properties through separate molecular pathways in a circuit-dependent manner. Specifically, Slitrk1 plays a key role in modulating spontaneous excitatory synaptic activity and NMDA receptor dynamics, whereas Slitrk2 is primarily involved in controlling asynchronous neurotransmitter release and synaptic response strength. The study further uncovers notable differences in their molecular interactions. While both Slitrk1 and Slitrk2 associate with LAR-RPTPs, Slitrk2 uniquely engages with PDZ domain-containing proteins and TrkB receptors, suggesting a broader role beyond trans-synaptic adhesion. These findings emphasize the specialized and non-overlapping contributions of Slitrk proteins in hippocampal synaptic organization, reinforcing the notion that cell adhesion molecules can execute highly specific functions within neural circuits.*

**We appreciate the reviewer’s overall positive assessment of our manuscript and their constructive suggestions for improving our original manuscript for publication. In the revised manuscript, we have done our best to fully address the reviewer’s comments.**

*1. A particularly intriguing aspect of this study is its investigation of the schizophrenia-associated Slitrk2 V89M mutation, which selectively impairs asynchronous excitatory transmission and leads to deficits in spatial reference memory. The observation that this mutation disrupts key aspects of synaptic function despite preserving surface expression, LAR-RPTP-binding activity, and synaptogenic properties suggests a pathophysiological mechanism distinct from a simple loss-of-function effect. This finding aligns with an emerging view in neuropsychiatric research that synaptic dysfunctions linked to mental disorders often manifest in circuit-specific and activity-dependent ways, rather than through a uniform reduction in synaptic density or adhesion. However, it is critical to verify that the expression levels of the mutant Slitrk2 protein remain comparable to wild-type (WT) following recombination. This type of conditional allele design can sometimes introduce unexpected changes in mRNA or protein expression, and the authors should confirm that Slitrk2 V89M is expressed at WT levels using quantitative protein and/or transcript analyses.*

**In Supplementary Figure 1 of the original manuscript, we provided data showing that Slitrk2-V89M protein is expressed in KI mice at levels comparable to those of Slitrk2 WT. As the reviewer asked whether KI mice have *Slitrk* mRNA expression levels similar to those of control mice, we performed qPCRs using hippocampal CA1 lysates of KI mice. We found that all five *Slitrk* mRNAs were expressed similarly in control and KI mice. These new results are presented in the Supplementary Figure 16 of the revised manuscript.**

*2. While the study provides valuable mechanistic insights into how Slitrk paralogs shape excitatory circuits, several aspects of the work remain unclear or require further validation. The epitope-preserving magnified analysis of proteome (eMAP) imaging, which was used to examine Slitrk1 and Slitrk2 distribution, raises some concerns regarding signal specificity and potential background noise. Given the relatively diffuse staining patterns observed, it remains uncertain whether the reported expression differences reflect true protein localization or artifacts of the technique. Additional knockout control staining is needed to ensure that the detected signals are not simply due to non-specific fluorescence. Moreover, while the authors propose that Slitrk1 and Slitrk2 differentially colocalize with VGLUT1 and PSD-95 in distinct hippocampal layers, these findings are not fully explored or rigorously quantified. Given that both proteins are known to be postsynaptic, their differential association with VGLUT1, a presynaptic marker, warrants further explanation.*

**We thank the reviewer for raising these important points regarding signal specificity, quantification, and interpretation. Regarding the eMAP imaging, autofluorescence is significantly reduced in eMAP gels compared to conventional paraformaldehyde-treated tissues due to optical clearing and volumetric dilution during gel preparation (e.g., PMID: 27454740). Nonetheless, we agree with the reviewer that the denaturation step performed during eMAP gelation could potentially damage the antibody-binding epitope, and thus the contribution of non-specific binding cannot be fully assessed without control staining of knockout mice. We addressed this critical concern by performing eMAP staining using the respective *Slitrk*-cKO tissues. Please see our detailed response to comment #6 of reviewer 1 for these data and the related discussion. Concerning the differential colocalization of Slitrk proteins with VGLUT1 versus PSD-95, we note that the eMAP tissues used in the current study underwent 4× linear expansion. The effective XY resolution limit with a 63 × objective is a biological distance of ~ 50 nm, and the resolution limit of the Z dimension is ~125 nm. Thus, a partial overlap between presynaptic VGLUT1 signals and Slitrk signals is indeed possible. To definitively address this concern, we removed the VGLUT1 staining from the colocalization experiments. Instead, we performed triple immunostaining for Slitrks and two established postsynaptic markers: SHANK2 and PSD-95. We rigorously quantified the colocalization of Slitrks with SHANK2 and PSD-95 and included these new results in Figure 1 and Supplementary Figure 2 of the revised manuscript.**

*3. Another concern is the variability in AAV-mediated Cre recombination efficiency, which could influence the extent of Slitrk deletion across animals. The study does not provide detailed quantification of viral spread or deletion efficiency, leaving open the possibility that differences in synaptic transmission may arise from incomplete recombination rather than intrinsic Slitrk function. The authors should include immunostaining or qPCR to precisely map the regions of effective gene deletion. This will ensure that the observed phenotypes are not an artifact of heterogeneous expression patterns.*

**The reviewer’s points are important and valid. We included immunofluorescence images from mouse brain sections infected with AAVs expressing Cre recombinase to show that there was accurate viral targeting and acceptable viral spread in the intended brain area. Although we had provided representative images showing that AAV-mediated Cre successfully deleted each Slitrk paralog in Figure S1 of the original manuscript, we now provide the relevant quantitative analyses showing that ~99% of neurons are infected with AAVs expressing ΔCre-GFP or Cre-GFP in adult mice. These new results are now presented in Supplementary Figure 3 of the revised manuscript.**

*4. The molecular interaction studies, while informative, also raise important unanswered questions. The claim that Slitrk1 and Slitrk2 require different intracellular pathways to mediate their functions is largely supported by in vitro experiments but lacks in vivo validation. Demonstrating that dominant-negative forms of LAR-RPTPs or TrkB can disrupt Slitrk-dependent synaptic effects in hippocampal neurons would provide stronger evidence that these pathways are functionally relevant in intact circuits. However, given the complexity of this approach, this is a recommendation but not binding on the authors to perform.*

**These are excellent suggestions. However, as the reviewer surmised, the interactions between Slitrks and their extracellular ligands (LAR-RPTPs or TrkB) do not depend on the phosphatase (for LAR-RPTPs) or kinase (for TrkB) activities of Slitrk ligands. Thus, even if dominant-negative constructs for LAR-RPTPs or TrkB could impair excitatory synaptic properties in specific hippocampal microcircuits, it would not be straightforward to interpret the ensuing phenotypes. We appreciate the reviewer’s suggestion but feel that this would be better addressed in follow-up studies.**

*Overall, this is a data-rich study based on mostly stringent and elegant experiments that appear to have been conducted carefully. The information on Slitrks provided is clearly important and of interest in the field of synapse biology. However, the study has limited conceptually advancement, insofar as the molecular mechanisms are concerned. Despite these limitations, the study makes a strong case for the functional diversification of Slitrk paralogs within hippocampal circuits. It underscores the importance of cell adhesion molecules in defining microcircuit properties, an area that remains underexplored in synaptic biology.*

**We again appreciate the reviewer’s overall positive assessment and constructive comments, which helped us improve the manuscript. We hope that our efforts made during the revision process render this study ready for publicationy in *PloS Biology.***

*Reviewer #3 : In this manuscript, Kim et al. showed that two similar synaptic CAMs are involved in different aspects of the specific wiring of the hippocampus, demonstrating that these CAMs are not redundant. Using conditional knockouts for Sltrk1 and Slitrk2, the authors show that synaptic specificity emerges in a context-dependent manner, likely depending on the proximity to different partners and intracellular signalling pathways. They also showed some links to schizophrenia. There is a manuscript with a very similar structure and partially similar findings showing the involvement of Slitrk1 in synaptic specificity (Schroeder et al., Neuron 2018). This paper further confirms those findings using a conditional knockout (the previous paper used a shRNA strategy) and compares them with another member of the same family (Slitrk2). Also, this paper adds a comparison with a second region, the dentate gyrus and shows that it is context-dependent. It is not full of novelty. However, the data is solid, and the amount of work provided by the authors is substantial, demonstrating additional roles for Slitrk2 and a link to neurodevelopmental disorders. There are some points that the authors need to address to strengthen the manuscript.*

**We appreciate the reviewer’s overall positive assessment, including their statement that “*the data is solid and the amount of work provided by the authors is substantial*”, and are grateful for the various constructive comments that helped us improve the manuscript.**

*Figure 1:*

*-What is the somatic expression of Slitrk1 and Slitrk2? Are the same neurons expressing both? The authors should run single-molecule in situ hybridization that can be quantified (assuming that the antibody staining will localize the protein at the synaptic terminals, not the soma). It is essential to consider whether different enrichments can explain part of the different functions.*

**We thank the reviewer for raising this important question regarding the somatic expression and coexpression of Slitrk1 and Slitrk2, and their suggestion that we perform quantitative single-molecule *in situ* hybridization (FISH). Unfortunately, it proved technically infeasible to perform a reliable quantitative analysis of mRNA expression using single-molecule FISH within the period of this revision. A commercially designed Slitrk2-specific probe proved inappropriate, as its signals were not suppressed in the hippocampal regions of adult *Slitrk2*-cKO mice (see Figure R1, which is included for the reviewer’s information). Moreover, Slitrk1 and Slitrk2 exhibit significant sequence homology, with most regions having less than 50 bp mismatch. This high homology means that commercially available probes often target regions with substantial overlap; this leads to a significant risk of cross-talk and prevents accurate determination of differential enrichment. Furthermore, even if specific probes were designed, the distance separating synapses from the neuronal soma means that it is inherently difficult to reliably correlate somatic mRNA expression patterns with synaptic protein expression. Although the results presented in Figure 1 of the original manuscript indicated some differential enrichment of Slitrk1 and Slitrk2 in different CA1 and DG layers, we agree that these patterns alone cannot fully account for the distinct phenotypes observed in hippocampal microcircuits. We hope that the reviewer accepts these explanations regarding the current technical limitations, and we intend to use these critical suggestions to guide future investigations into Slitrk expression patterns.**

**Figure R1. Analyses of RNAscope-based distribution of *Slitrk2* mRNAs in adult mouse hippocampal CA1 sections. Note that *Slitrk2* mRNAs are still detected in *Slitrk2*-cKO CA1 neurons, leading us to conclude that the commercial Slitrk2-specific probe is not appropriate for this purpose**

*-The expression of Slitrk1 in Figure 1K and 1 J is challenging to visualize. Would it be possible to improve the signal (e.g. increase brightness, and of course, at equal levels on Slitrk2)? It is difficult to conclude the quantifications in Figures 1L and 1M. Are Slitrk1 and 2 either presynaptic or postsynaptic proteins? A schema in the figure to the findings may help. Also, it will be more informative to identify the synapses by colocalising VGLUT1 and PSD95 and then co-localizing with Slitrk1 and 2, with this method or another, to confirm the localization at the synapses.*

**We appreciate the reviewer’s suggestions on how we could improve original Figures 1K and 1J (now presented in Supplementary Figure 2A–2D). To enhance visualization and facilitate comparison, we adjusted the contrast of the images, using identical settings for each hippocampal area. We agree that it is important to clarify the synaptic expression patterns of Slitrk1 and Slitrk2. As these proteins are primarily associated with postsynaptic sites, we performed new experiments using triple immunostaining with the established postsynaptic markers, SHANK2 and PSD-95. We removed the VGLUT1-related images from revised Figure 1 to avoid ambiguity with respect to presynaptic markers. SHANK2 and PSD-95 are both excitatory postsynaptic markers; they exhibit a high degree of colocalization and allowed us to clearly assess and quantify the synaptic expression patterns of Slitrk1 and Slitrk2. Revised Figure 1 now includes these colocalization data, with representative images presented in Supplementary Figure 2.**

*Figure 4:*

*-Since the infected cells are not identified in the electron microscopy experiments, it will be important to show the levels of infection the authors reach with the viral experiments. The ratio of infected cells should be provided.*

**This comment echoes comment #3 of reviewer #2. To address this point, we performed immunohistochemical analyses demonstrating that ~95% of hippocampal CA1 neurons were infected by the AAVs and ~100% of infected neurons exhibited suppression of the target Slitrk proteins.**

*- The count of docked vesicles in Slitrk1 conditional mutants decreases (Figure 4B and C). However, the frequency of sEPSCs increases (Figure 2C and D). How can the authors explain this? It seems counterintuitive, given that the sEPSCs frequency usually reflects the number of synapses and release probability. What is the density of synapses in pyramidal cells in these experiments?*

**This question echoes comment #1 of reviewer #1. We agree that the indicated electron microscopic and electrophysiological phenotypes seem contradictory. However, the number of docked vesicles does not necessarily correlate with spontaneous synaptic transmission or synapse number *per se* because the TEM data reflects the ultrastructural changes in each synapse. Thus, we performed immunohistochemical analyses using adult *Slitrk1*-cKO and *Slitrk2*-cKO mice. The results revealed that loss of Slitrk1 or Slitrk2 in the CA1 or DG decreases the excitatory synapse number, as assessed by antibodies against excitatory synaptic markers (anti-VGLUT1 and anti-PSD-95). These new results are presented in Supplementary Figure 8 of the revised manuscript. We also note that the frequency of sEPSCs might be highly correlated with the neurotransmitter release probability, but not necessarily with the synapse number. Thus, we performed whole-cell patch clamp recordings to measure mEPSCs, which better reflect the synapse number. Consistent with our immunohistochemical results, we found that the mEPSC frequency was reduced in *Slitrk1*-cKO mice. These new results are presented in Supplementary Figure 9 of the revised manuscript. Since we previously reported that *Slitrk2*-cKO in the hippocampal CA1 exhibits decreased the mEPSC frequency (PMID: 35840571), we did not repeat this experiment in the revised manuscript. We hope that these new data fully resolve the reviewer’s concerns.**

*Supplementary Figures:*

*-To assess redundancy, the authors conduct electrophysiology in the double Slitrk1cKO;Slitrk2cKO in the dentate gyrus (in Fig. S5). Is there any reason why they do not run the experiments in CA1? If any, this is more relevant since it is the core area in the manuscript; the dentate gyrus is used to show other regions.*

**We appreciate the reviewer’s careful observation. There was particular rationale for omitting an analysis of CA1 pyramidal neurons from *Slitrk1/2* double-cKO mice from the original manuscript. To fill this gap, we performed extensive new analyses using *Slitrk1/2* double-cKO mice. The results showed that Slitrk1 and Slitrk2 are also functionally non-redundant in the CA1, as seen in the DG. These new results are presented in Supplementary Figure 11 of the revised manuscript.**

*-In Fig. S1F, Slitrk1fl/fl/Slitrk2f/f double cKO show a reduction in Gephyrin and PSD95 protein levels. The single cKO does not have a reduction in these proteins. Does this reflect a reduction in excitatory and inhibitory synapses in this model? Could the authors explain this to you?*

**We appreciate the reviewer’s careful observation. At present, we are unable to provide a convincing response to this important question. It is possible that simultaneous deletion of Slitrk1 and Slitrk2 together impacts inhibitory synapse maintenance. However, this was not investigated in the current study and we believe that it is beyond the scope of the current study. To avoid introducing confusion, we removed the gephyrin and PSD-95 immunoblots from the revised manuscript.**

*Minor*

*-Page 15 "**stsynaptic"*

**We corrected this typographic error in the revised manuscript.**

**Again, we thank the reviewers for their careful assessment of our paper and hope that the revised manuscript is now acceptable for publication in *Plos Biology.***

**----------------------------------------------------------------------------------------------------------**

**Bibliographical references**

Cho WH, Noh K, Lee BH, Barcelon E, Jun SB, Park HY, Lee SJ (2022) Hippocampal astrocytes modulate anxiety-like behavior. *Nat Commun* 13: 6536

Ku T, Swaney J, Park JY, Albanese A, Murray E, Cho JH, Park YG, Mangena V, Chen J, Chung K (2016) Multiplexd and scalable super-resolution imaging of three-dimensional protein localization in size-adjustable tissues. *Nat Bioctech* 34: 973-981

Luo F, Südhof TC (2017) Synaptotagmin-7-Mediated Asynchronous Release Boosts High-Fidelity Synchronous Transmission at a Central Synapse. *Neuron* 94: 826-839

Sando R, Südhof TC (2021) Latrophilin GPCR signaling mediates synapse formation. *Elife* 10: e65717

Sclip A, Südhof TC (2023) Combinatorial expression of neurexins and LAR-type phosphotyrosine phosphatase receptors instructs assembly of a cerebellar circuit. *Nat Commun* 14: 4976

Williamson MR, Kwon W, Woo J, Ko Y, Maleki E, Yu K, Murali S, Sardar D, Deneen B (2025) Learning-associated astrocyte ensembles regulate memory recall. *Nature* 637: 478-486
