# Supplementary material for: Paralogs of Slitrk cell adhesion molecules configure excitatory synapse specificity via distinct cellular mechanisms
Source: PLoS Biol. 2025 Dec 18;23(12):e3003576. doi: 10.1371/journal.pbio.3003576 (PMC12742733; doi:10.1371/journal.pbio.3003576)
Supplement: Attachment — Submitted filename: Rebuttal_revision [FINAL].doc [file pbio.3003576.s027.doc]

**Authors’ rebuttal letter for Kim, Kim and Kim *et al*., “Excitatory Slitrk paralogs configure synaptic specificity via distinct extracellular and intracellular mechanisms”, and description of changes made to the revised manuscript**

**We are grateful to the reviewers for their careful evaluation and critical analysis of our manuscript. Our responses to the specific comments of the reviewers are provided below, with the complete reviewers’ comments shown in *italic* typeface, our response in bold typeface, and the cited text in bold blue typeface.**

**Note from the Editorial Office**

*1. A 'Response to Reviewers' file - this should detail your responses to the editorial requests, present a point-by-point response to all of the reviewers' comments, and indicate the changes made to the manuscript.*

**NOTE: In your point-by-point response to the reviewers, please provide the full context of each review. Do not selectively quote paragraphs or sentences to reply to. The entire set of reviewer comments should be present in full and each specific point should be responded to individually, point by point. You should also cite any additional relevant literature that has been published since the original submission and mention any additional citations in your response.*

**We followed the instructions provided by the Editorial Office by tailoring our rebuttal letter to provide detailed responses to the full text of each comment and indicate relevant changes in the revised manuscript.**

*2. In addition to a clean copy of the manuscript, please also upload a 'track-changes' version of your manuscript that specifies the edits made. This should be uploaded as a "Revised Article with Changes Highlighted" file type.*

**As instructed, we uploaded both changes-tracked and clean copies of the manuscript.**

**Re-submission Checklist**

*When you are ready to resubmit your revised manuscript, please refer to this re-submission checklist:* [*https://plos.io/Biology_Checklist*](https://plos.io/Biology_Checklist)

*To submit a revised version of your manuscript, please go to* [*https://www.editorialmanager.com/pbiology/*](https://www.editorialmanager.com/pbiology/) *and log in as an Author. Click the link labelled 'Submissions Needing Revision' where you will find your submission record.*

**We referred to this checklist and ensured that all materials were uploaded.**

*Please make sure to read the following important policies and guidelines while preparing your revision:*

**Published Peer Review**

*Please note while forming your response, if your article is accepted, you may have the opportunity to make the peer review history publicly available. The record will include editor decision letters (with reviews) and your responses to reviewer comments. If eligible, we will contact you to opt in or out. Please see here for more details:* [*https://blogs.plos.org/plos/2019/05/plos-journals-now-open-for-published-peer-review/*](https://blogs.plos.org/plos/2019/05/plos-journals-now-open-for-published-peer-review/)

**PLOS Data Policy**

*Please note that as a condition of publication PLOS' data policy (*[*http://journals.plos.org/plosbiology/s/data-availability*](http://journals.plos.org/plosbiology/s/data-availability)*) requires that you make available all data used to draw the conclusions arrived at in your manuscript. If you have not already done so, you must include any data used in your manuscript either in appropriate repositories, within the body of the manuscript, or as supporting information (N.B. this includes any numerical values that were used to generate graphs, histograms etc.). For an example see here: [http://www.plosbiology.org/article/info%3Adoi%2F10.1371%2Fjournal.pbio.1001908#s5](http://www.plosbiology.org/article/info%3Adoi%2F10.1371%2Fjournal.pbio.1001908#s5)
*Blot and Gel Data Policy**

*We require the original, uncropped and minimally adjusted images supporting all blot and gel results reported in an article's figures or Supporting Information files. We will require these files before a manuscript can be accepted so please prepare them now, if you have not already uploaded them. Please carefully read our guidelines for how to prepare and upload this data: [https://journals.plos.org/plosbiology/s/figures#loc-blot-and-gel-reporting-requirements](https://journals.plos.org/plosbiology/s/figures#loc-blot-and-gel-reporting-requirements)
*Protocols deposition**

*To enhance the reproducibility of your results, we recommend that if applicable you deposit your laboratory protocols in* [*protocols.io*](http://protocols.io/)*, where a protocol can be assigned its own identifier (DOI) such that it can be cited independently in the future. Additionally, PLOS ONE offers an option for publishing peer-reviewed Lab Protocol articles, which describe protocols hosted on* [*protocols.io*](http://protocols.io/)*. Read more information on sharing protocols at* [*https://plos.org/protocols?utm_medium=editorial-email&utm_source=authorletters&utm_campaign=protocols*](https://plos.org/protocols?utm_medium=editorial-email&utm_source=authorletters&utm_campaign=protocols)

**We reviewed the various policies of PLOS Biology and followed them in the revised manuscript.**

**Reviewers' comments:**

*Reviewer #1: While the authors can be commended for the DREADD experiment, which supports that the loss of Slitrks affects sEPSCs independent of their effect on intrinsic excitability, key concerns remain. These concerns apply to data in the original study and new results presented in this revision.*

**We thank the reviewer for providing several constructive comments and hope that the revised manuscript addresses their remaining concerns.**

*Major points*

*1. The selective removal of data from the original manuscript that had been criticized in the original submission raises questions. This applies to the VGlut1 eMAP data, which we raised questions about in point #12. We were surprised to find these VGLUT1-Slitrk colocalization data removed from Fig. 1 without any acknowledgement or rationale provided to the reviewer, especially given the authors' response to our point #12 in our review. The Gephyrin and PSD95 blots, which reviewer #3 questioned, were also removed. It is unclear why the authors removed these data instead of keeping them in and performing additional experiments or simply stating these findings as-is in the text.*

**We apologize for the confusion and concern caused by our failure to clearly explan these changes. We assure the reviewer that we did not intend to hide data. These decisions were made to improve the manuscript’s flow and focus on our strongest supporting evidence. Upon initial reassessment, including the reviewer’s previous valid concerns (point #12 of the first round of review), we concluded that the VGLUT1-Slitrk colocalization data were quantitatively less robust in supporting our claims than the other evidence we provided. We decided that the newly fortified synapse quantification results presented in S8 Fig in the revised manuscript provide a much more direct and powerful demonstration of the structural synaptic changes seen following Slitrk loss. Thus, we decided to remove the VGLUT1 eMAP data, which we deemed redundant and comparatively less clear. During this round of revision, upon additional review of the Slitrk puncta number between control and cKO groups, it was determined that the Slitrk2 antibody used for eMAP experiments was not suitable. Due to the lack of change in the total number of Slitrk2^+^ puncta, we removed the relevant results from Fig 1. In the Results section, we now clearly describe the failure of the Slitrk2 antibodies used for eMAP experiments. We have also included the Slitrk1^+^ and Slitrk2^+^ puncta results in S2 Fig for the purpose of transparency. Regarding our removal of the gephyrin and PSD-95 blots from S1 Fig, this was done to address a point made by Reviwer #3. We also acknowledged that the data raised important questions concerning inhibitory synapses that we were, at present, “unable to provide a convincing response to” and noted that a full investigation into this was “beyond the scope of the current study”. Reviewer #3 found this explanation reasonable and was satisfied with this approach. Our intention was not to obscure data, but rather to remove preliminary findings that raised complex questions we could not adequately address within the current study, thereby keeping the manuscript focused on the robust excitatory synapse data that we can fully support. We hope that this clarifies that these omissions were made for rational reasons of clarity and scope and were accepted by the reviewer who initially raised the point.**

*2. The synapse quantifications are a critical aspect of this work. However, the pattern of immunostaining are not compelling, and the extent of change in synapse number in Fig. S8 raises questions. Based on the representative images, VGLUT1 and PSD95 do not exhibit the punctate appearance expected for this region and do not appear to colocalize well, which is expected for these two markers of excitatory synapses. How can the authors ensure this immunostaining is suitable for synapse quantification? The lack of detail provided on synapse quantification methods is not helpful, either. Using these stainings, the authors describe an ≥80% drop in synapse number in select layers of the KO hippocampus. This is very surprising, as the deletion of most synaptic proteins results in no or only modest changes in synapse number, with alpha-Neurexins being one of many examples.  It should be added that such a large drop in synapse number would not align with the more modest drop in mEPSC frequency in Fig. S9.*

**We thank the reviewer for these critical questions. We agree on the importance of the synapse quantification and have thoroughly addressed the reviewer’s concerns regarding (1) staining visualization, (2) methodological detail, and (3) the discrepancy between morphological and functional data, as follows. (1) Staining: We acknowledge that our original images presented in S8 Fig, which were processed primarily to show overall density differences, unfortunately obscured the distinct, punctate nature of the individual signals and their colocalization. To directly address this, we reprocessed and generated new representative images that are now shown in S8 Fig of the revised manuscript. We have explicitly marked the colocalized puncta with arrows to visually depict the signals that were quantified as *bona fide* excitatory synapses. We are confident that these new and improved visualizations, combined with the detailed methodological description discussed below, fully resolve the reviewer’s concerns regarding the suitability of our staining for quantification. We also affirm that the utilized antibodies were extensively validated for this application in previous literature and that our staining protocol followed established standards. (2) Detailed methodology: We have significantly expanded the Methods section to provide a comprehensive description of our protocol. This includes detailed image acquisition parameters, and a precise description of the image processing and analysis workflow, including the specific thresholding method and the explicit criteria used to define a colocalized synaptic punctum. (3) The apparent discrepancy between morphological and functional data: The reviewer correctly pointed out the apparent discrepancy between the ~80% reduction in morphological synapse number (S8 Fig) and the more modest decrease in mEPSC frequency (S9 Fig). We propose that this is not a contradiction, but rather a key biological insight of the current study. We suggest that the two techniques may be sampling distinct synapse populations. The mEPSC recordings are from whole-cell patches on CA1 pyramidal neuron somata, and thus capture spontaneous events from a subset of synapses impinging on these cells. In contrast, the imaging quantification is a layer-specific analysis of ALL VGLUT1/PSD-95 puncta within that volume, which includes synapses onto interneurons or different dendritic compartments that may be differentially affected by *Slitrk*-cKO. It is also equally plausible that homeostatic compensation mechanism was activated by a large-scale structural loss of excitatory synapses in the surviving synaptic population. We have added a new paragraph to the Discussion section to thoroughly discuss this finding and its potential biological implications.**

*Minor points*

*3. The use of knockout controls for the eMAP experiments is important. However, some of the representative images shown are not convincing in showing specificity.*

**We thank the reviewer for this critical feedback. We agree that the specificity of our eMAP experiments should be unambiguously clear in the representative images. Our original intention was to provide a broad, representative view of the signal distribution across the analyzed subfield, rather than focusing on a few select synapses. However, we acknowledge the reviewer’s point that this approach, combined with potential resolution loss during publication formatting, may have obscured the fine details required to convincingly demonstrate specificity. We believe that the modified presentation and text now provide both the representative overview we intended and the detailed evidence of specificity that the reviewer rightly requested. We have also ensured that all images are submitted at the highest possible resolution to minimize data loss.**

*4. When quantifying Slitrk synaptic puncta in the conditional knockouts via eMAP, the authors should include an analysis of the total number and average size of Slitrk puncta to confirm whether the total amount of Slitrks has been diminished in the conditional knockouts.*

**We appreciate the reviewer’s constructive suggestion that we validate our cKO models via eMAP quantification. We agree that the average puncta size would be a valuable metric. However, accurate 3D puncta segmentation for size analysis requires high-resolution imaging with very fine Z-axis step sizes, which is unfortunately beyond the capabilities of our current imaging equipment. Thus, we focused on the total number of puncta, as suggested. Following the reviewer’s suggestion, we quantified the total number of Slitrk puncta. This analysis was successful for Slitrk1, as we observed a significant reduction in the number of Slitrk1 puncta in *Slitrk1*-cKO samples compared to controls, which provided eMAP confirmation of the successful KO at the protein level. We now include this quantification data in S2 Fig. However, when we performed the same analysis for Slitrk2, we failed to observe the expected decrease in puncta number in *Slitrk2*-cKO samples, even though a parallel analysis confirmed that Cre recombinase was efficiently expressed in the *Slitrk2*-floxed mice. This discrepancy led us to conclude that the Slitrk2 antibody, while suitable for other applications, likely produces non-specific (false-positive) signals in eMAP-processed tissues and is thus unsuitable for this specific quantification. To address the reviewer’s critical feedback and ensure the accuracy of our manuscript, we removed all eMAP-based quantitative data and claims related to Slitrk2 from the revised manuscript, including the related data in former Fig 1 and S2. We also now clearly explain this technical limitation. We rely on the other methods already presented in the paper to confirm the knockout *Slitrk2*. We believe that by providing the successful Slitrk1 eMAP quantification and transparently removing the non-specific Slitrk2 eMAP data, we have appropriately addressed the reviewer’s concern and improved the overall robustness of our study. We again thank the reviewer for highlighting this issue, which prevented the inclusion of potentially misleading results.**

*5. In reference to point #5 in our initial review, it would be beneficial for the authors to explicitly state the degree of baseline variability in the electrophysiology data in the text of the manuscript. This transparency would aid readers in assessing the findings of the manuscript.*

**We agree that we need to be transparent with the readers about the variability in baseline values observed between our different control groups across experiments, not just the stability within a single recording. To address this directly, we added a clarifying statement to the Methods section as follows:** *We noted that baseline electrophysiological values (e.g., mEPSC frequency) for control groups exhibited some variability between independent experiments. This degree of variation is commonly observed in the field for such complex recordings and, in our study, may also have been influenced by the necessary use of different AAV-ΔCre-injected transgenic lines as respective controls for different conditional knockout experiments. Therefore, to ensure valid comparisons and account for this inter-experiment variability, all data within a given experimental set were first normalized to the mean of their respective internal control group before statistical comparisons were made across conditions.*

*6. In response to point #14 in our initial review of the manuscript, we noted a contradiction in the discussion of Slitrk redundancy. The authors' revised sentence still bears this contradiction: "This suggests that Slitrk1 and Slitrk2 are functionally non-redundant in regulating various excitatory synaptic properties in the hippocampal DG microcircuits, but likely operate via convergent pathways in regulating basal synaptic transmission and intrinsic excitability in CA1 pyramidal neurons, as shown by the opposite phenotypes produced by loss of each Slitrk paralog". As "operating via convergent pathways" implies molecular redundancy, it is unclear how this statement could be true.*

**We thank the reviewer for catching our continued poor choice of wording. The reviewer is correct that “convergent pathways” carries the implication of redundancy, which is the opposite of what we intended to convey and, as the reviewer rightly noted, directly contradicts our “non-redundant” claim based on the “opposite phenotypes”. We apologize for the repeated confusion on this point. Our intention was not to imply that Slitrk1 and Slitrk2 converge on a single redundant pathway. Rather, we meant to state that these two non-redundant paralogs, through their distinct molecular mechanisms, both ultimately act to modulate the same set of physiological properties (basal transmission and intrinsic excitability), but they do so in opposing directions. To eliminate this contradiction entirely, we corrected this confusing sentence in the Discussion section, as follows:** *These results reveal that opposite phenotypes are produced by loss of each Slitrk paralog, suggesting that Slitrk1 and Slitrk2 are functionally non-redundant in regulating various excitatory synaptic properties in the hippocampal CA1 and DG microcircuits; instead, they act via distinct and opposing mechanisms to regulate basal synaptic transmission and intrinsic excitability in CA1 pyramidal neurons, as clearly demonstrated by the opposite phenotypes produced by the loss of each Slitrk paralog.* **We believe this revision finally resolves the discrepancy and accurately reflects our observations and the implications of the opposite phenotypes.**

*7. The authors should be more upfront about the somewhat selective application of several experimental designs in this study. For instance, the authors should explicitly state that the Barnes Maze test and several electrophysiological analyses were performed for the CA1 circuits, but not the dentate gyrus circuit in the Slitrk2 V89M mutant. This limits the extent to which this study supports the circuit-specific effects of this mutant.*

**We appreciate the reviewer’s comment, but we wish to clarify that this was a deliberate, rationale-driven experimental design, not an arbitrary limitation. As we explained in our previous response, our specific goal was to test a highly focused hypothesis based on our prior work. In our previous study (PMID: 35840571), we definitively established that the conditional deletion of Slitrk2 specifically in CA1 neurons results in a deficit of spatial reference memory, which was specifically identified using the Barnes Maze test (no other behavioral abnormality was detected). Thus, the objective of the current experiment was to examine whether the pathogenic Slitrk2 V89M mutant *recapitulates* this known, CA1-specific, Barnes Maze-dependent behavioral phenotype. We agree with the reviewer that this specific rationale was not made sufficiently clear to the reader. To correct this and provide full transparency, we now explicitly explain this in the Results section, where the Barnes Maze data is introduced, as follows:** *We previously showed that hippocampal CA1-specific deletion of Slitrk2 impairs spatial reference memory; this was assessed by the Barnes maze test and represented the sole identified behavioral deficit associated with the CA1-specific cKO of Slitrk2 [17]. Here, we set out to directly test the hypothesis that the pathogenic Slitrk2 V89M will recapitulate this known CA1-dependent behavioral phenotype. We thus examined…* **This text clarifies that this specific circuit and test were chosen to allow us to directly link our current findings to our previous, published results. We hope that this action assuages the reviewer’s concern.**

*8. The authors continue to make very strong statements about their results. Several points in our review of the initial manuscript asked the authors to tone down their interpretations of this data.*

**We have carefully reviewed the entire manuscript, paying special attention to the Abstract and Discussion sections, to ensure that our interpretations do not overstate the data. We have done our best to ensure that all conclusions are strictly supported by the presented data.**

*Reviewer #2: The authors have addressed all my concerns satisfactorily.*

**We thank Reviewer 2 for this positive assessment.**

*Reviewer #3: The authors have addressed most of my concerns. The data is robust, and the extent of work presented by the authors is impressive, illustrating additional roles for Slitrk2 and a connection to neurodevelopmental disorders.*

**We are grateful to Reviewer 3 for his/her appraisal of our revised manuscript and for highlighting its significance.**

**Again, we thank the reviewers for their careful assessment of our paper and hope that the revised manuscript is now acceptable for publication in *PLOS Biology.***
